# World catalogue of the genus-group names in Elateridae (Insecta, Coleoptera). Part I: Agrypninae, Campyloxeninae, Hemiopinae, Lissominae, Oestodinae, Parablacinae, Physodactylinae, Pityobiinae, Subprotelaterinae, Tetralobinae

**DOI:** 10.3897/zookeys.839.33279

**Published:** 2019-04-16

**Authors:** Robin Kundrata, Magdalena Kubaczkova, Alexander S. Prosvirov, Hume B. Douglas, Anna Fojtikova, Cleide Costa, Yves Bousquet, Miguel A. Alonso-Zarazaga, Patrice Bouchard

**Affiliations:** 1 Department of Zoology, Faculty of Science, Palacky University, 17. listopadu 50, 77146, Olomouc, Czech Republic Palacky University Olomouc Czech Republic; 2 Department of Entomology, Faculty of Biology, Moscow State University, Leninskie gory 1/12, 119234, Moscow, Russia Moscow State University Moscow Russia; 3 Canadian National Collection of Insects, Arachnids and Nematodes, Agriculture and Agri-Food Canada, 960 Carling Avenue, Ottawa, Ontario, K1A 0C6, Canada Agriculture and Agri-Food Canada Ottawa Canada; 4 Museu de Zoologia, Universidade de São Paulo, Caixa Postal 42.494, CEP 04218-970, São Paulo, SP, Brazil Universidade de São Paulo São Paulo Brazil; 5 Gatineau, Québec, Canada Unaffiliated Ottawa Canada; 6 Depto. de Biodiversidad y Biología Evolutiva, Museo Nacional de Ciencias Naturales (CSIC), C/. José Gutiérrez Abascal, 2, E-28006 Madrid, Spain Museo Nacional de Ciencias Naturales Madrid Spain

**Keywords:** Classification, click-beetles, Elateroidea, new combinations, new genera, nomenclature, Staphylinidae, systematics

## Abstract

In this first part of the World catalogue of genus-group names in Elateridae, a nomenclatural review of the genera belonging to ten subfamilies is provided. All names are given with author name, year, and page of publication, type species, and type fixation. We list 132 valid genera in Agrypninae, 2 in Campyloxeninae, 4 in Hemiopinae, 11 in Lissominae, 2 in Oestodinae, 8 in Parablacinae, 2 in Physodactylinae, 2 in Pityobiinae, 1 in Subprotelaterinae, and 7 in Tetralobinae. Genera *Anathesis* Candèze, 1865, *Antitypus* Candèze, 1882, *Chrostus* Candèze, 1878, *Dorygonus* Candèze, 1859 (with subgenus Rygodonus Fleutiaux, 1932), and *Macromalocera* Hope, 1834 are tentatively placed as Agrypninae*incertae sedis*. *Paradrapetesvillosus* Fleutiaux, 1895 is designated as the type species for *Paradrapetes* Fleutiaux, 1895. Two new genera are proposed based on species previously incorrectly used as type species for *Abiphis* Fleutiaux, 1926 and *Lycoreus* Candèze, 1857. These genera are *Neoabiphis* Kundrata & Bouchard, **gen. n.** (type species: *Elaternobilis* Illiger, 1800) and *Neolycoreus* Kundrata & Bouchard, **gen. n.** (type species: *L.regalis* Candèze, 1857), respectively. The following new combinations are proposed for species hitherto included in *Abiphis* Fleutiaux, 1926: *Neoabiphiscandezei* (Alluaud, 1896), **comb. n.**, *N.fairmairei* (Fleutiaux, 1903), **comb. n.**, *N.goudoti* (Fleutiaux, 1942), **comb. n.**, *N.insignis* (Klug, 1833), **comb. n.**, *N.nobilis* (Illiger, 1800), **comb. n.**, and *N.viettei* (Girard, 1966), **comb. n.** The following new combinations are proposed for species hitherto included in *Lycoreus* Candèze, 1857: *Neolycoreusalluaudi* (Candèze, 1900), **comb. n.**, *N.corpulentus* (Candèze, 1899), **comb. n.**, *N.cyclops* (Candèze, 1865), **comb. n.**, *N.decorsei* (Fleutiaux, 1903), **comb. n.**, *N.dux* (Candèze, 1857), **comb. n.**, *N.goudotii* (Laporte, 1838), **comb. n.**, *N.madagascariensis* (Gory, 1832), **comb. n.**, *N.oculipennis* (Fairmaire, 1903), **comb. n.**, *N.orbiculatus* (Schwarz, 1901), **comb. n.**, *N.regalis* (Candèze, 1857), **comb. n.**, *N.sicardi* (Fleutiaux, 1942), **comb. n.**, *N.triangularis* (Fleutiaux, 1942), **comb. n.**, *N.triocellatus* (Laporte, 1838), **comb. n.**, and *N.vicinus* (Fleutiaux, 1942), **comb. n.** The following new combinations are proposed for species hitherto incorrectly included in *Plectrosternus* Lacordaire, 1857: *Legnarufa* (Lacordaire, 1857), **comb. n.**, *L.convexa* (Vats, 1991), **comb. n.**, *L.coolsi* (Schimmel, 1996), **comb. n.**, and *L.foveata* (Patwardhan & Athalye, 2012), **comb. n.** This research revealed a nomenclatural problem threatening the stability of the well-established valid genus name *Adelocera* Latreille, 1829. An application to the International Commission on Zoological Nomenclature will be necessary in this case to maintain stability. Additionally, we act here as First Revisers (ICZN 1999, Art. 24.2) in giving precedence to *Lucarius* Gistel, 1848 (Staphylinidae) over *Lucarius* Gistel, 1848 (Elateridae).

## Introduction

Elateridae, commonly known as click-beetles, is a large beetle family containing approximately 10,000 described species worldwide (Costa et al. 2010). Although they are well-known and usually easy-to-recognise as a family, even for non-specialists, the limits of, as well as phylogenetic interrelationships within, Elateridae are still in flux (e.g., Lawrence and Newton 1995; Costa et al. 2010; Douglas 2011; Kundrata and Bocak 2011; Kundrata et al. 2016, 2018a; Douglas et al. 2018; Bocak et al. 2018). Various suprageneric classifications have been proposed for the family, and also generic limits often considerably vary between different authors (e.g., Fleutiaux 1947a; Hayek 1973; Gurjeva 1974a, b; Dolin 1975; Stibick 1979; Calder 1996; Sánchez-Ruiz 1996; Cate et al. 2007; Costa et al. 2010; Kundrata et al. 2018a). Although recent catalogues are available for some world regions (e.g., Golbach 1994, Calder 1996, Cate et al. 2007, Arias-Bohart and Elgueta 2012, Zapata and Sánchez-Ruiz 2012, Bousquet et al. 2013, Aguirre-Tapiero and Johnson 2014, Johnson and Chaboo 2015, Platia and Ghahari 2016, Girard 2017, Tarnawski et al. 2018) or for certain suprageneric groups (e.g., Schimmel 1999, 2003, 2004, 2005; Tarnawski 1996, 2001; Schimmel et al. 2015, 2016; Schimmel and Tarnawski 2009, 2010, 2012; Douglas 2017; Kubaczkova and Kundrata 2017; Kundrata et al. 2018b, c), a comprehensive catalogue of the world Elateridae fauna is not available. Therefore, many recent studies on Elateridae still follow works by Candèze (1857, 1859, 1860, 1863, 1891), Schwarz (1906a, 1907), and Schenkling (1925, 1927). Those authors, however, did not include data on the type species and type fixation in their catalogues. The study of diversity, taxonomy, phylogeny, and biogeography of Elateridae is not possible without genus-group name concepts which are consistent with regulations from the International Commission on Zoological Nomenclature (ICZN 1999). The first attempt to cover all genus-group names in Elateridae was by Hyslop (1921), followed by additions to that catalogue by Arnett (1955). However, despite their great effort, these authors introduced some nomenclatural inconsistencies, and new supraspecific names in Elateridae have been proposed since that time. Therefore, it is necessary to update the information on genus-group names for Elateridae, and correct these errors still commonly repeated by contemporary authors.

By compiling this catalogue, we work to provide a summary of all genus-group names including synonymies, misspellings, and their availability in the sense of the International Code of Zoological Nomenclature (ICZN 1999). We also indicate the type species for all genera including information on their designations, give the systematic position of each genus according to the most recent studies, and provide a robust framework for future Elateridae workers. This catalogue could serve as a starting point for future Elateridae-related studies, not only focused on systematics but also on diversity, evolution, biogeography, and nature conservation.

## Materials and methods

We compiled all genus-group names in the Elateridae subfamilies Agrypninae, Campyloxeninae, Hemiopinae, Lissominae, Oestodinae, Parablacinae, Physodactylinae, Pityobiinae, and Tetralobinae. Genus-group names in Elaterinae, Thylacosterninae, Morostomatinae, Plastocerinae, Negastriinae, Cardiophorinae and Dendrometrinae sensu lato (including e.g., Hypnoidini, Oxynopterini, Semiotini, Dimini and Senodoniini) will be treated in the next parts of the catalogue. The family classification follows the Handbook of Zoology (Costa et al. 2010), with additional changes proposed by Bouchard et al. (2011; family-group names), Kundrata and Bocak (2011, several classification changes based on the molecular phylogeny), Schimmel and Tarnawski (2012, description of the tribe Tetrigusina), Kundrata et al. (2016, description of the subfamily Parablacinae), and Kundrata et al. (2018a, subfamilial rank proposed for Tetralobinae). A synopsis of this suprageneric classification of extant Elateridae is given in Table 2 in Kundrata et al. (2018a).

The generic compositions of the tribes and subfamilies treated in this study are as follows: Agrypnini sensu Hayek (1973, 1979, as Agrypninae, but excluding *Lanelater* Arnett, 1952), with genera added by Chassain (1983), Gurjeva (1987) and Arias-Bohart (2014), and changes by Gurjeva (1977), Cate et al. (2007), and Girard (2017); Anaissini sensu Golbach (1984), including Alampina of Costa (1975); Euplinthini sensu Costa (1975), with changes by Johnson (2002b) and Arias-Bohart and Elgueta (2012); Drilini sensu Kundrata and Bocak (2011, 2017); Hemirhipini sensu Casari (2008; but excluding *Anthracalaus* Fairmaire, 1888), with changes by Patwardhan et al. (2009), Schimmel and Tarnawski (2012), and Arimoto and Arimoto (2017); Oophorini sensu Johnson (1995, 1997) and Cate et al. (2007), with changes by Calder (1996), Platia (2007, 2012), and Girard (2017); Pseudomelanactini sensu Johnson (2002a); Pyrophorini sensu Costa (1975), plus *Metapyrophorus* Rosa & Costa, 2009; Campyloxeninae sensu Costa (1975), plus *Malalcahuello* Arias-Bohart, 2015; Hemiopinae sensu Lawrence et al. (1999), with an additional genus transferred there by Douglas (2011); Lissominae sensu Lawrence and Arias (2009) and Arias-Bohart (2013), but excluding Oestodinae (see Kundrata et al. 2016); Oestodinae sensu Johnson (2002a; as Oestodini) and Kundrata et al. (2016); Parablacinae sensu Kundrata et al. (2016), plus *Sharon* Arias-Bohart & Elgueta, 2015; Physodactylinae sensu Rosa (2014); Pityobiinae sensu Kundrata et al. (2016); Subprotelaterinae sensu Costa et al. (2010), and Tetralobinae sensu Costa et al. (1994) and Kubaczkova and Kundrata (2017). Only extant taxa are included in the catalogue.

The names of the family-, genus- and species-group taxa are given with the name of the author, and the year and page of publication. The page given is the page where the taxon name and description are printed, except for the generic names first introduced in catalogues and made available by including one or more available species names (ICZN 1999, Art. 12.2.5). When a work was published in various parts we determined the oldest part in which a particular name became available for the first time, following the Principle of Priority (ICZN 1999, Art. 23.1). A search for each genus-group name was performed in the hundreds of publications dealing with Elateridae and its relatives, including all works cited here but also numerous other taxonomic revisions, regional faunistic studies, and checklists, general entomological books, etc. Additionally, we checked the online version of the Nomenclator Zoologicus (http://www.ubio.org/NomenclatorZoologicus/) to look for potentially important information on authorship, dates of publication and homonyms. The year and page given for the incorrect subsequent spellings are the first year and page in which they are used. Incorrect subsequent spellings not in prevailing usage are unavailable (ICZN 1999, Art. 33.3). Complete data and comments for family- and genus-group names are presented with the lowest-rank name (e.g., tribe rather than subfamily, subgenus not genus), since these criteria follow the Principle of Coordination (ICZN 1999, Art. 36.1 and 43.1). The detailed information for family-group names is given in Bouchard et al. (2011) and is not repeated here. We provide the type species for each genus-group name, including information on its designation (i.e., original designation, monotypy, and in the case of subsequent designation we provide author, year and page). New type species designations are proposed as needed to preserve taxonomic stability (ICZN 1999, Art. 69). We do not repeat type species and their fixations for replacement names as “both the prior nominal taxon and its replacement have the same type species, and type fixation for either applies also to the other, despite any statement to the contrary” (ICZN 1999, Art. 67.8). Under each family- and genus-group name, the currently valid name is listed the first, followed by synonyms in chronological order. Misspellings, unavailable names, and corrected stem formations are followed by colon “:”. Dates of publications and exact bibliographic references (especially problematic ones, often not cited uniformly by researchers) are taken from the following comprehensive general works: Evenhuis (1997), Cate et al. (2007), Bouchard et al. (2011), Bousquet and Bouchard (2013, 2017), Nagel and Schmidlin (2014), and Bousquet (2016).

## Catalogue

### Family Elateridae Leach, 1815

Elaterides Leach, 1815: 85. Type genus: *Elater* Linnaeus, 1758. As pointed out by several authors, e.g., Lawrence and Newton (1995) and Bouchard et al. (2011), the oldest name for this family is Cebrionidae Latreille, 1802. In 2011, a formal application to maintain usage of Elateridae over Cebrionidae was submitted by PJ Johnson for publication in the Bulletin of Zoological Nomenclature, and for an eventual vote by the International Commission on Zoological Nomenclature (ICZN). A notice for the reception of this new Case was published in December 2011 (ICZN 2011). The ICZN later closed this Case without further comments (ICZN 2012). Based on reviews by three ICZN Commissioners it had been decided that the Case is covered by Article 35.5 of the International Code of Zoological Nomenclature (ICZN 1999), and that no further actions were needed (ICZN Secretariat, personal communication). The same reasoning supports conserving usage of Elateroidea Leach, 1815 over Cebrionoidea Latreille, 1802.

Elateriadae: Arnett et al. 1969: 9 [unavailable name, incorrect subsequent spelling not in prevailing usage].

#### Subfamily Agrypninae Candèze, 1857

Agrypnides Candèze, 1857: 17. Type genus: *Agrypnus* Eschscholtz, 1829. The *nomen protectum*Agrypninae Candèze, 1857 was given precedence for subfamily name over the *nomen oblitum*Oophorinae Gistel, 1848 (ICZN 1999, Art. 35.5) by Bouchard et al. (2011).

##### Tribe Agrypnini Candèze, 1857

Adeloceraeidae Gistel, 1848a: [5]. Type genus: *Adelocera* Latreille, 1829. *Nomen oblitum*, incorrect original stem formation, not in prevailing usage; see Bouchard et al. (2011).

Pangauradae Gistel, 1856: 366. Type genus: *Pangaura* Gistel, 1856 (syn. of *Lacon* Laporte, 1838). *Nomen oblitum*, incorrect original stem formation, not in prevailing usage; see Bouchard et al. (2011).

Agrypnides Candèze, 1857: 17. Type genus: *Agrypnus* Eschscholtz, 1829. *Nomen protectum*; see Bouchard et al. (2011).

Octocryptites: Candèze 189﻿2: 486 [unavailable name, see Bouchard et al. 2011].

Adelocerini Buysson, 1893: 15. Type genus: *Adelocera* Latreille, 1829. Family-group name proposed as new without reference to Adeloceridae Gistel, 1848.

Octocryptini Schwarz, 1906a: 31. Type genus: *Octocryptus* Candèze, 1892.

Octocrytinae: Fleutiaux 1927a: 99 [incorrect stem formation (ICZN 1999, Art. 29.3)].

Agripninae: Miwa 1927: 12 [incorrect stem formation (ICZN 1999, Art. 29.3)].

Cavicoxumidae Pic, 1928: 21. Type genus: *Cavicoxum* Pic, 1928 (syn. of *Agraeus* Candèze, 1857). Incorrect original stem formation, not in prevailing usage.

Cavicoxidae: Arnett 1962: 497. Correct stem formation of Cavicoxumidae Pic, 1928.

Laconini Dajoz, 1964: 60. Type genus: *Lacon* Laporte, 1838.

Pangauridae: Lawrence and Newton 1995: 854. Correct stem formation of Pangauradae Gistel, 1856.

###### Genus *Acrocryptus* Candèze, 1874

*Cryptotarsus* Philippi, 1873: 308. Gender: masculine. Type species: *Cryptotarsusater* Philippi, 1873: 308, by monotypy. Preoccupied by *Cryptotarsus* Kirsch, 1865: 88 [Coleoptera: Melyridae: Malachiinae].

*Acrocryptus* Candèze, 1874: 39. Gender: masculine. Replacement name for *Cryptotarsus* Philippi, 1873.

*Hexaulacus* Candèze, 1874: 40. Gender: masculine. Type species: *Hexaulacusreedi* Candèze, 1874: 40, by monotypy. Synonymised by Golbach (1970: 307).

###### Genus *Adelocera* Latreille, 1829

*Adelocera* Latreille, 1829: 451. Gender: feminine. Type species: The widely accepted type species for this genus is *Elaterovalis* Germar, 1823: 49, by subsequent designation (Hyslop 1921: 623). However the valid type species is *Elaterfuscus* Fabricius, 1801: 228 (non Schrank, 1781, nec Geoffroy, 1785), by subsequent designation (Duponchel 1840: 119). Since *Elaterfuscus* Fabricius, 1801 is currently placed in the genus *Agrypnus* Eschscholtz, 1829, an application asking the International Commission on Zoological Nomenclature to set aside the earlier type species designation by Duponchel is necessary to conserve *Adelocera* Latreille, 1829 as the valid name of this genus.

*Pericus* Candèze, 1857: 167. Gender: masculine. Type species: *Pericusnitidus* Candèze, 1857: 167, by monotypy. Synonymised with *Adelocera* Latreille, 1829 by Hayek (1973: 13).

*Brachylacon* Motschulsky, 1858: 60. Gender: masculine. Type species: *Brachylaconmicrocephalus* Motschulsky, 1858: 60, by monotypy. Synonymised with *Adelocera* Latreille, 1829 by Hayek (1973: 13).

*Adolocera*: Candèze 1860: 110 [unavailable name, incorrect subsequent spelling not in prevailing usage].

*Prolacon* Fleutiaux, 1934a: 179. Gender: masculine. Type species: *Prolaconalluaudi*Fleutiaux 1934a: 179, by monotypy. Synonymised with *Adelocera* Latreille, 1829 by Hayek (1973: 13).

*Aganolacon* Ôhira, 1967a: 55. Gender: masculine. Type species: *Aganolaconshirozui* Ôhira, 1967a: 55, by original designation. Synonymised with *Adelocera* Latreille, 1829 by Hayek (1973: 13).

*Brachylason*: Dolin 1978: 6 [unavailable name, incorrect subsequent spelling not in prevailing usage].

*Adellocera*: Uniyal and Vats 1988: 49 [unavailable name, incorrect subsequent spelling not in prevailing usage].

###### Genus *Agraeus* Candèze, 1857

*Agraeus* Candèze, 1857: 165. Gender: masculine. Type species: *Agraeusmannerheimii* Candèze, 1857: 166, by monotypy.

*Trachylacon* Motschulsky, 1858: 60. Gender: masculine. Type species: *Trachylaconfulvicollis*Motschulsky 1858: 61, by subsequent designation (Hyslop 1921: 672). Synonymised with *Agraeus* Candèze, 1857 by Schwarz (1906a: 27).

*Trochylacon*: Motschulsky 1858: 61 [unavailable name, incorrect original spelling (ICZN 1999, Art. 19.3); First Reviser (ICZN 1999, Art. 24.2): Motschulsky (1869: 34)].

*Cavicoxum* Pic, 1928: 21. Gender: neuter. Type species: *Cavicoxummonstrosum* Pic, 1928: 21, by monotypy. Synonymised with *Agraeus* Candèze, 1857 by Fleutiaux (1931: 74).

###### Genus *Agrypnus* Eschscholtz, 1829

*Agrypnus* Eschscholtz, 1829: 32. Gender: masculine. Type species: *Elatermurinus* Linnaeus, 1758: 406, by subsequent designation (Curtis 1838: 694), see Sánchez-Ruiz (1996: 43).

*Mecynocanthus* Hope, 1837: 53. Gender: masculine. Type species: *Mecynocanthusunicolor* Hope, 1837: 53, by monotypy. Synonymised with *Agrypnus* Eschscholtz, 1829 by Hayek (1973: 113).

*Agripnus*: Laporte 1838: 10 [unavailable name, incorrect subsequent spelling not in prevailing usage].

*Tylotarsus* Germar, 1840: 247. Gender: masculine. Type species: *Tylotarsuscinctipes* Germar, 1840: 248, by monotypy. Synonymised with *Agrypnus* Eschscholtz, 1829 by Hayek (1973: 113).

*Tilotarsus*: Candèze 1857: 86 [unavailable name, incorrect subsequent spelling not in prevailing usage].

*Myrmodes* Candèze, 1857: 168. Gender: masculine. Type species: *Myrmodesakidiformis* Candèze, 1857: 169, by monotypy. Synonymised with *Agrypnus* Eschscholtz, 1829 by Hayek (1973: 113).

*Archontas* Gozis, 1886: 23. Gender: masculine. Type species: *Elatermurinus* Linnaeus, 1758: 406, by monotypy. Junior objective synonym of *Agrypnus* Eschscholtz, 1829.

*Pseudolacon* Blackburn, 1890: 89. Gender: masculine. Type species: *Pseudolaconrufus* Blackburn, 1890: 90, by monotypy. Synonymised with *Agrypnus* Eschscholtz, 1829 by Hayek (1973: 113).

*Homoeolacon* Blackburn, 1890: 90. Gender: masculine. Type species: *Homoeolacongracilis* Blackburn,1890: 91, by monotypy. Synonymised with *Agrypnus* Eschscholtz, 1829 by Hayek (1973: 113).

*Enoploderes* Schwarz, 1898a: 131. Gender: masculine. Type species: *Elatercuspidatus* Klug, 1833: 66, by monotypy. Preoccupied by *Enoploderes* Faldermann, 1837: 309 [Coleoptera: Cerambycidae].

*Centrostethus* Schwarz, 1898b: 414. Gender: masculine. Replacement name for *Enoploderes* Schwarz, 1898a. Synonymised with *Agrypnus* Eschscholtz, 1829 by Hayek (1973: 113).

*Paralacon* Reitter, 1905: 6. Gender: masculine. Type species: *Laconcinnamomeus* Candèze, 1874: 76, by monotypy. Synonymised with *Agrypnus* Eschscholtz, 1829 by Hayek (1973: 113).

*Centhrostethus*: Hyslop 1921: 634 [unavailable name, incorrect subsequent spelling not in prevailing usage].

*Aprypnus*: Winkler and Buysson in Winkler, 1925: 578 [unavailable name, incorrect subsequent spelling not in prevailing usage].

*Tyloarsus*: Fleutiaux 1934b: 59 [unavailable name, incorrect subsequent spelling not in prevailing usage].

*Colaulon* Arnett, 1952: 116. Gender: masculine. Type species: *Elaterrectangularis* Say, 1825: 263, by original designation. Synonymised with *Agrypnus* Eschscholtz, 1829 by Hayek (1973: 113).

*Agryphus*: Arnett 1955: 611 [unavailable name, incorrect subsequent spelling not in prevailing usage].

*Cryptolacon* Nakane & Kishii, 1955: 1. Gender: masculine. Type species: *Cryptolaconmiyamotoi* Nakane & Kishii, 1955: 1, by original designation. Synonymised with *Agrypnus* Eschscholtz, 1829 by Hayek (1973: 113).

*Sabikikorius* Nakane & Kishii, 1955: 3. Gender: masculine. Type species: *Laconfuliginosus* Candèze, 1865: 10, by original designation. Synonymised with *Agrypnus* Eschscholtz, 1829 by Hayek (1973: 113).

*Sagojyo* Kishii, 1964: 30. Gender: masculine. Type species: Colaulon (Sagojyo) yuppe Kishii, 1964: 31, by original designation. Synonymised with *Agrypnus* Eschscholtz, 1829 by Hayek (1973: 113).

*Archontoides* Cobos, 1966: 651. Gender: masculine. Type species: *Archontoidespretoriensis* Cobos, 1966: 652, by monotypy. Synonymised with *Agrypnus* Eschscholtz, 1829 by Hayek (1973: 113).

*Pyrganus* Golbach, 1968: 198. Gender: masculine. Type species: *Lacontuspanensis* Candèze, 1857: 157, by original designation. Synonymised with *Agrypnus* Eschscholtz, 1829 by Hayek (1973: 113).

*Sagojo*: Ôhira 1968: 364 [unavailable name, incorrect subsequent spelling not in prevailing usage].

*Homeolacon*: Hayek 1973: 113. [unavailable name, incorrect subsequent spelling not in prevailing usage].

###### Genus *Candanius* Hayek, 1973

*Anius* Candèze, 1889: 103. Gender: masculine. Type species: *Aniusgracillimus* Candèze, 1889: 103, by monotypy. Preoccupied by *Anius* Pascoe, 1885: 312 [Coleoptera: Curculionidae].

*Candanius* Hayek, 1973: 85. Gender: masculine. Replacement name for *Anius* Candèze, 1889.

###### Genus *Carlota* Arias-Bohart, 2014

*Carlota* Arias-Bohart, 2014: 59. Gender: feminine. Type species: *Carlotacoigue* Arias-Bohart, 2014: 60, by original designation.

###### Genus *Christinea* Gurjeva, 1987

*Christinea* Gurjeva, 1987: 39. Gender: feminine. Type species: *Christineamirabilis* Gurjeva, 1987: 39, by original designation.

###### Genus *Compsolacon* Reitter, 1905

*Compsolacon* Reitter, 1905: 6. Gender: masculine. Type species: *Elatercrenicollis* Ménétriés, 1832: 156, by monotypy. Removed from synonymy with *Agrypnus* Eschscholtz, 1829 by Gurjeva (1977: 793); generic status confirmed by Prosvirov and Savitsky (2011: 760).

*Neolacon* Miwa, 1929: 234. Gender: masculine. Type species: *Neolaconformosanus* Miwa, 1929: 235, by original designation. Synonymised with *Compsolacon* Reitter, 1905 by Miwa (1934: 14).

*Nealacon*: Miwa 1930: 91 [unavailable name, incorrect subsequent spelling not in prevailing usage].

*Campsolacon*: Gurjeva 1974b: 72 [unavailable name, incorrect subsequent spelling not in prevailing usage].

###### Genus *Compresselater* Platia & Gudenzi, 2006

*Compresselater* Platia & Gudenzi, 2006: 132. Gender: masculine. Type species: *Compresselaterbicolor* Platia & Gudenzi, 2006: 133, by original designation.

###### Genus *Danosoma* C.G. Thomson, 1859

*Danosoma* C.G. Thomson, 1859: 103. Gender: neuter. Type species: *Elaterconspersus* Gyllenhal, 1808: 377, by original designation.

*Delox*: Quelle 1932: 208 [unavailable name, no type species originally designated (ICZN 1999, Art. 13.3); not made available subsequently, Arnett (1955: 607) listed it in synonymy with *Danosoma* CG Thomson, 1859].

*Danasoma*: Gülperçin and Tezcan 2010: 2 [unavailable name, incorrect subsequent spelling not in prevailing usage].

###### Genus *Dilobitarsus* Latreille, 1834

*Dilobitarsus* Latreille, 1834: 142. Gender: masculine. Type species: *Dilobitarsustuberculatus* Latreille, 1834: 143 (syn. of *Elaterbidens* Fabricius, 1801: 227), by monotypy.

*Dilobotarsus* Agassiz, 1846: 124. Gender: masculine. Unjustified emendation of *Dilobitarsus* Latreille, 1834, not in prevailing usage.

*Anacantha* Solier, 1851: 18. Gender: feminine. Type species: *Anacanthasulcicollis* Solier, 1851: 18, by original designation. Synonymised with *Dilobitarsus* Latreille, 1834 by Hayek (1973: 93).

###### Genus *Eidolus* Candèze, 1857

*Eidolus* Candèze, 1857: 178. Gender: masculine. Type species: *Eidoluslinearis* Candèze, 1857: 179, by monotypy.

###### Genus *Elasmosomus* Schwarz, 1902

*Elasmosomus* Schwarz, 1902a: 212. Gender: masculine. Type species: *Elasmosomusfasciculatus* Schwarz, 1902a: 214, by subsequent designation (Hyslop 1921: 643).

###### Genus *Hemicleus* Candèze, 1857

*Hemicleus* Candèze, 1857: 180. Gender: masculine. Type species: *Hemicleuscaffer* Candèze, 1857: 181, by monotypy.

###### Genus *Lacon* Laporte, 1838

*Lepidotus* Stephens, 1830: 374. Gender: masculine. Type species: *Elatervarius* Olivier, 1790: 32 (syn. of *Elaterquerceus* Herbst, 1784: 113), by subsequent designation (Hyslop 1921: 652). Preoccupied by *Lepidotus* Asso, 1801: 38 [Pisces].

*Lacon* Laporte, 1838: 11. Gender masculine (^1^). Type species: *Elateratomarius* Fabricius, 1798: 139 (syn. of *Elaterpunctatus* Herbst, 1784: 110), by subsequent designation (Hyslop 1921: 652).

*Pangaura* Gistel, 1856: 366. Gender: feminine. Type species: *Elatervarius* Olivier, 1790: 32 (syn. of *Elaterquerceus* Herbst, 1784: 113), by Sánchez-Ruiz (1996: 44). Senior objective synonym of *Zalepia* Arnett, 1953.

*Ocneus* Candèze, 1857: 84. Gender: masculine. Type species: *Ocneuslimbatus* Candèze, 1857: 85, by monotypy. Synonymised with *Lacon* Laporte, 1838 by Hayek (1973: 52).

*Ochneus*: Desmarest 1860: 24 [unavailable name, incorrect subsequent spelling not in prevailing usage].

*Scelisus* Candèze, 1863: 327. Gender: masculine. Type species: *Scelisussanguineus* Candèze, 1863: 328, by monotypy. Synonymised with *Lacon* Laporte, 1838 by Hayek (1973: 52).

*Alaotypus* Schwarz, 1902b: 307. Gender: masculine.Type species: *Alaotypussubpectinatus* Schwarz, 1902b: 308, by subsequent designation (Hyslop 1921: 625). Synonymised with *Adelocera* Latreille, 1829 (sensu auct., = *Lacon* Laporte, 1838) by Fleutiaux (1918b: 183); syn. of *Lacon* Laporte, 1838 (see Hayek 1973: 52).

*Aulacon* Arnett, 1952: 112. Gender: masculine. Type species: *Adeloceranobilis* Fall, 1932: 58, by original designation. Synonymised with *Lacon* Laporte, 1838 by Hayek (1973: 53).

*Diphyaulon* Arnett, 1952: 111. Gender: neuter. Type species: *Adelocerapyrsolepis* LeConte, 1866: 389, by original designation. Synonymised with *Lacon* Laporte, 1838 by Hayek (1973: 53).

*Zalepia* Arnett, 1953: 7. Gender: feminine. Replacement name for *Lepidotus* Stephens, 1830. Synonymised with *Lacon* Laporte, 1838 by Hayek (1973: 53). Junior objective synonym of *Pangaura* Gistel, 1856.

*Kobulacon* Chûjô & Ôhira, 1965: 2. Gender: masculine. Type species: *Laconquadrinodatus* Lewis, 1894: 28, by original designation. Synonymised with *Lacon* Laporte, 1838 by Hayek (1973: 53).

*Arnettia* Golbach, 1969b: 155. Gender: feminine. Type species: *Adeloceraaberrans* Candèze, 1874: 23, by monotypy. Synonymised with *Lacon* Laporte, 1838 by Hayek (1973: 53).

*Cornilacon* Golbach, 1969b: 158. Gender: masculine. Type species: *Adeloceralongicornis* Champion, 1894: 261, by original designation. Synonymised with *Lacon* Laporte, 1838 by Hayek (1973: 53).

*Latilacon* Golbach, 1969b: 158. Gender: masculine. Type species: *Adeloceralaticollis* Candèze, 1857: 59, by original designation. Synonymised with *Lacon* Laporte, 1838 by Hayek (1973: 53).

*Monocyrton* Golbach, 1969b: 156. Gender: neuter. Type species: *Adelocerachabannii* Guérin-Méneville, 1829: plate 12, by original designation. Synonymised with *Lacon* Laporte, 1838 by Hayek (1973: 53).

*Lepidelater* Smith in Arnett et al. 1969: 11. Gender: masculine. Type species: *Lepidelatermisticius* Mignot in Arnett et al. 1969: 12, by original designation. Synonymised with *Lacon* Laporte, 1838 by Hayek (1973: 53).

*Lason*: Dolin 1978: 6 [unavailable name, incorrect subsequent spelling not in prevailing usage].

*Alaeotypus*: Carpenter 1992: 305 [unavailable name, incorrect subsequent spelling not in prevailing usage].

###### Genus *Lobotarsus* Schwarz, 1898

*Lobotarsus* Schwarz, 1898a: 131. Gender: masculine. Type species: *Lobotarsusdecoratus* Schwarz, 1898a: 131, by subsequent designation (Hyslop 1921: 653). Synonymised with *Agrypnus* Eschscholtz, 1829 by Hayek (1973: 113) but removed from synonymy by Girard (2017: 27).

*Lobitarsus*: Fleutiaux 1935: 93 [unavailable name, incorrect subsequent spelling not in prevailing usage].

###### Genus *Meristhus* Candèze, 1857

*Meristhus* Candèze, 1857: 162. Gender: masculine. Type species: *Elaterlepidotus* Palisot de Beauvois, 1805: 11, by subsequent designation (Desmarest 1860: 24).

####### Subgenus Meristhus Candèze, 1857

*Meristhus* Candèze, 1857: 162. Gender: masculine. Type species: *Elaterlepidotus* Palisot de Beauvois, 1805: 11, by subsequent designation (Desmarest 1860: 24).

*Rhaciaspis* Arnett, 1952: 121. Gender: feminine. Type species: *Elaterlepidotus* Palisot de Beauvois, 1805: 11, by original designation. Junior objective synonym of *Meristhus* Candèze, 1857.

*Rhasciapis*: Golbach 1969a: 141 [unavailable name, incorrect subsequent spelling not in prevailing usage].

*Meristus*: Dolin 1975: 1625 [unavailable name, incorrect subsequent spelling not in prevailing usage].

####### Subgenus Sulcimerus Arnett, 1955

*Sulcimerus*: Fleutiaux 1947a: 255 [unavailable name, no type species originally designated (ICZN 1999, Art. 13.3)].

*Sulcimerus* Arnett, 1955: 617. Gender: masculine. Type species: *Meristhusquadripunctatus* Candèze, 1857: 163, by original designation.

###### Genus *Octocryptus* Candèze, 1892

*Octocryptus* Candèze, 1892: 486. Gender: masculine. Type species: *Octocryptuscardoni* Candèze, 1892: 487, by monotypy.

*Octorcryptus*: Gurjeva 1974b: 72 [unavailable name, incorrect subsequent spelling not in prevailing usage].

###### Genus *Optaleus* Candèze, 1857

*Optaleus* Candèze, 1857: 86. Gender: masculine. Type species: *Optaleuslimbatus* Candèze, 1857: 87, by subsequent designation (Hyslop 1921: 660).

*Opatelus*: Hayek 1973: 6 [unavailable name, incorrect subsequent spelling not in prevailing usage].

###### Genus *Rismethus* Fleutiaux, 1947

*Rismethus* Fleutiaux, 1947a: 257. Gender: masculine. Type species: *Meristhusscobinula* Candèze, 1857: 164, by original designation.

###### Genus *Saudilacon* Chassain, 1983

*Saudilacon* Chassain, 1983: 138. Gender: masculine. Type species: *Saudilaconochraceus* Chassain, 1983: 138, by original designation.

###### Genus *Scaphoderus* Candèze, 1857

*Scaphoderus* Candèze, 1857: 46. Gender: masculine. Type species: *Scaphoderusriehlii* Candèze, 1857: 46, by monotypy.

*Bruyantius* Fleutiaux, 1925: 101. Gender: masculine. Type species: *Bruyantiuscapensis* Fleutiaux, 1925: 102, by monotypy. Synonymised with *Scaphoderus* Candèze, 1857 by Hayek (1973: 51).

###### Genus *Stangellus* Golbach, 1975

*Stangellus* Golbach, 1975: 256. Gender: masculine. Type species: *Stangellusbucheri* Golbach, 1975: 257, by monotypy.

###### Genus *Sulcilacon* Fleutiaux, 1927

*Sulcilacon* Fleutiaux, 1927a: 65. Gender: masculine. Type species: *Adelocerageographica* Candèze, 1865: 7, by original designation. Synonymised with *Lacon* Laporte, 1838 by Hayek (1973: 52) but removed from synonymy by Girard (2017: 7).

###### Genus *Trieres* Candèze, 1900

*Trieres* Candèze, 1900: 78. Gender feminine (^2^). Type species: *Trieresramitarsus* Candèze, 1900: 78, by monotypy.

*Triers*: Hayek 1973: 7, 239 [unavailable name, incorrect subsequent spelling not in prevailing usage].

##### Tribe Anaissini Golbach, 1984

Alampina Costa, 1975: 54. Type genus: *Alampes* Champion, 1896 [preoccupied genus name, not *Alampes* Horváth, 1884 [Hemiptera: Lygaeidae]]; permanently invalid (Art. 39): based on preoccupied type genus.

Anaissini Golbach, 1984: 81. Type genus: *Anaissus* Candèze, 1857.

###### Genus *Agnostelater* Costa, 1975

*Agnostelater* Costa, 1975: 55. Gender: masculine. Type species: *Pyrophorusmesochrous* Germar, 1843: 91, by original designation.

###### Genus *Alampoides* Schwarz, 1906a

*Alampoides* Schwarz, 1906a: 216. Gender: masculine. Type species: *Pyrophorussubmaculatus* Schwarz, 1902a: 287, by subsequent designation (Hyslop 1921: 625).

###### Genus *Anaissus* Candèze, 1857

*Anaissus* Candèze, 1857: 187. Gender: masculine. Type species: *Anaissustarsalis* Candèze, 1857: 188, by monotypy.

####### Subgenus Anaissus Candèze, 1857

*Anaissus* Candèze, 1857: 187. Gender: masculine. Type species: *Anaissustarsalis* Candèze, 1857: 188, by monotypy.

####### Subgenus Auctumnalis Calder, 1978

*Auctumnalis* Calder, 1978: 303. Gender: masculine. Type species: *Anaissusaurantium* Calder, 1978: 303, by original designation.

###### Genus *Coctilelater* Costa, 1975

*Coctilelater* Costa, 1975: 64. Gender: masculine. Type species: *Alampescorymbitoides* Candèze, 1900: 95, by original designation.

###### Genus *Peralampes* Johnson, 2002

*Alampes* Champion, 1896: 474. Gender: masculine. Type species: *Alampesvestitus* Champion, 1896: 474, by subsequent designation (Hyslop 1921: 624). Preoccupied by *Alampes* Horváth, 1884: 10 [Hemiptera: Lygaeidae].

*Peralampes* Johnson, 2002b: 16. Gender: masculine. Replacement name for *Alampes* Champion, 1896.

##### Tribe Euplinthini Costa, 1975

Euplinthina Costa, 1975: 66. Type genus: *Euplinthus* Costa, 1975. Bouchard et al. (2011) acted as First Revisers (ICZN 1999, Art. 24.2) (Compsoplinthina Costa, 1975 vs. Euplinthina Costa, 1975)].

###### Subtribe Cleidecostina Johnson, 2002

Heligmini Costa, 1975: 53. Type genus: *Heligmus* Candèze, 1865 [preoccupied genus name, not *Heligmus* Dujardin, 1845 [Nematoda]]; permanently invalid (ICZN 1999, Art. 39): based on preoccupied type genus (see Bouchard et al. 2011).

Cleidecostini Johnson, 2002b: 16. Type genus: *Cleidecosta* Johnson, 2002. Replacement name for Heligmini Costa, 1975 because of the homonymy of the type genus.

####### Genus *Cleidecosta* Johnson, 2002

*Heligmus* Candèze, 1865: 52. Gender: masculine. Type species: *Heligmusglyphoderus* Candèze, 1865: 52, by monotypy. Preoccupied by *Heligmus* Dujardin, 1845: 147 [Nematoda].

*Cleidecosta* Johnson, 2002b: 16. Gender: feminine. Replacement name for *Heligmus* Candèze, 1865.

####### Genus *Meroplinthus* Candèze, 1891

*Meroplinthus* Candèze, 1891: 163. Gender: masculine. Type species: *Pyrophorustrinotatus* Candèze, 1882: 91, by subsequent designation (Hyslop 1921: 657).

*Meorplinthus*: Gurjeva 1974b: 72 [unavailable name, incorrect subsequent spelling not in prevailing usage].

####### Genus *Pyrischius* Hyslop, 1921

*Ischius* Candèze, 1857: 195. Gender: masculine. Type species: *Ischiusgerstaeckeri* Candèze, 1857: 196, by monotypy. Preoccupied by *Ischius* Wesmael, 1837: 20 [Hymenoptera].

*Pyrischius* Hyslop, 1921: 668. Gender: masculine. Replacement name for *Ischius* Candèze, 1857.

###### Subtribe Compsoplinthina Costa, 1975

Compsoplinthina Costa, 1975: 71. Type genus: *Compsoplinthus* Costa, 1975.

####### Genus *Compsoplinthus* Costa, 1975

*Compsoplinthus* Costa, 1975: 71. Gender: masculine. Type species: *Pyrophorusruber* Candèze, 1878b: clxix, by original designation.

###### Subtribe Euplinthina Costa, 1975

Euplinthina Costa, 1975: 66. Type genus: *Euplinthus* Costa, 1975.

####### Genus *Arcanelater* Costa, 1975

*Arcanelater* Costa, 1975: 66. Gender: masculine. Type species: *Pyrophorusspurius* Germar, 1841: 56, by original designation.

####### Genus *Euplinthus* Costa, 1975

*Euplinthus* Costa, 1975: 67. Gender: masculine. Type species: *Monocrepidiusophthalmicus* Perty, 1830: 21, by original designation.

####### Genus *Paraphileus* Candèze, 1882

*Paraphileus* Candèze, 1882: 92. Gender: masculine. Type species: *Aphanobiusthoreyi* Germar, 1844: 188, by monotypy.

##### Tribe Drilini Blanchard, 1845

Drilites Blanchard, 1845: 53. Type genus: *Drilus* Olivier, 1790.

###### Genus *Drilus* Olivier, 1790

*Drilus* Olivier, 1790: [23] 1. Gender: masculine. Type species: *Ptilinusflavescens* Geoffroy, 1785: 4, by monotypy.

*Cochleoctonus* Mielzinsky, 1824: 74. Gender: masculine. Type species: *Cochleoctonusvorax* Mielzinsky, 1824: 75, by monotypy. Synonymised with *Drilus* Olivier, 1790 by Desmarest (1824: 268).

###### Genus *Flabelloselasia* Kundrata & Bocak, 2017

*Flabelloselasia* Kundrata & Bocak, 2017: 443. Gender: feminine. Type species: *Flabelloselasiaoculata* Kundrata & Bocak, 2017: 445, by original designation.

###### Genus *Kupeselasia* Kundrata & Bocak, 2017

*Kupeselasia* Kundrata & Bocak, 2017: 448. Gender: feminine. Type species: *Kupeselasiaminuta* Kundrata & Bocak, 2017: 450, by original designation.

###### Genus *Lolosia* Kundrata & Bocak, 2017

*Lolosia* Kundrata & Bocak, 2017: 452. Gender: feminine. Type species: *Lolosiatransversalis* Kundrata & Bocak, 2017: 454, by original designation.

###### Genus *Malacogaster* Bassi, 1834

*Malacogaster* Bassi, 1834: pl. 99. Gender: feminine. Type species: *Malacogasterpasserinii* Bassi, 1834: pl. 99, by monotypy.

###### Genus *Microselasia* Kundrata & Bocak, 2017

*Microselasia* Kundrata & Bocak, 2017: 455. Gender: feminine. Type species: *Microselasiaobscura* Kundrata & Bocak, 2017: 467, by original designation.

###### Genus *Selasia* Laporte, 1838

*Selasia* Laporte, 1838: 19. Gender: feminine. Type species: *Selasiarhipiceroides* Laporte, 1838: 20, by monotypy.

###### Genus *Wittmerselasia* Kundrata & Bocak, 2017

*Wittmerselasia* Kundrata & Bocak, 2017: 470. Gender: feminine. Type species: *Wittmerselasiacamerooniana* Kundrata & Bocak, 2017: 473, by original designation.

####### Subgenus Wittmerselasia Kundrata & Bocak, 2017

*Wittmerselasia* Kundrata & Bocak, 2017: 470. Gender: feminine. Type species: *Wittmerselasiacamerooniana* Kundrata & Bocak, 2017: 473, by original designation.

####### Subgenus Latoselasia Kundrata & Bocak, 2017

*Latoselasia* Kundrata & Bocak, 2017: 481. Gender: feminine. Type species: *Latoselasiasimilis* Kundrata & Bocak, 2017: 483, by original designation.

##### Tribe Hemirhipini Candèze, 1857

Hémirhipides Candèze, 1857: 199. Type genus: *Hemirhipus* Berthold, 1827.

###### Subtribe Hemirhipina Candèze, 1857

Hémirhipides Candèze, 1857: 199. Type genus: *Hemirhipus* Berthold, 1827. First Reviser (ICZN 1999, Art. 24.2) found (Hemirhipini Candèze, 1857 vs. Chalcolepidiini Candèze, 1857) is Casari-Chen (1985: 392).

Chalcolépidiides Candèze, 1857: 257. Type genus: *Chalcolepidius* Eschscholtz, 1829.

Alaites Candèze, 1874: 112. Type genus: *Alaus* Eschscholtz, 1829.

Chalcolepidina: Leng 1920: 167 [incorrect stem formation (ICZN 1999, Art. 29.3)].

Hemirrhipininae: Miwa 1927: 12 [incorrect stem formation (ICZN 1999, Art. 29.3)].

Alauinae Laurent, 1974: 16. Type genus: *Alaus* Eschscholtz, 1829 [proposed as new without reference to Alaites Candèze, 1874; incorrect original stem formation, not in prevailing usage].

####### Genus *Alaolacon* Candèze, 1865

*Alaolacon* Candèze, 1865: 13. Gender: masculine. Type species: *Alaolaconcyanipennis* Candèze, 1865: 13, by monotypy.

*Eumoeus* Candèze, 1874: 113. Gender: masculine. Type species: *Eumoeusmurrayi* Candèze, 1874: 113, by original designation. Synonymised with *Alaolacon* Candèze, 1865 by Arimoto & Arimoto (2017: 88).

*Luzonicus* Fleutiaux, 1916a: 232. Gender: masculine. Type species: *Luzonicusbakeri* Fleutiaux, 1916a: 232, by monotypy. Synonymised with *Eumoeus* Candèze, 1874 by Fleutiaux (1947a: 306).

*Tharopsides* Fleutiaux, 1918a: 235. Gender: masculine. Type species: *Tharopsidesharmandi* Fleutiaux, 1918a: 235, by subsequent designation (Hyslop 1921: 671). Synonymised with *Eumoeus* by Fleutiaux (1928b: 178).

*Eumaeus*: Fleutiaux 1941b: 40 [unavailable name, incorrect subsequent spelling not in prevailing usage.

####### Genus *Alaomorphus* Hauser, 1900

*Alaomorphus* Hauser, 1900: 141. Gender: masculine. Type species: *Alaomorphuscandezei* Hauser, 1900: 143, by monotypy.

####### Genus *Alaus* Eschscholtz, 1829

*Alaus* Eschscholtz, 1829: 33. Gender: masculine. Type species: *Elateroculatus* Linnaeus, 1758: 404, by subsequent designation (Duponchel 1840: 242).

*Lamprias* Gistel, 1834: 11. Gender: masculine. Type species: *Elateroculatus* Linnaeus, 1758: 404, by monotypy. Preoccupied by *Lamprias* Bonelli, 1810: Tabula Synoptica [Coleoptera: Carabidae]. Junior objective synonym of *Alaus* Eschscholtz, 1829. Unnecesarily synonymised with *Alaus* Eschscholtz, 1829 by Bousquet & Bouchard (2017: 124).

*Alans*: Desmarest 1860: 25 [unavailable name, incorrect subsequent spelling not in prevailing usage].

*Olaüs*: Fairmaire 1878: 279 [unavailable name, incorrect subsequent spelling not in prevailing usage].

####### Genus *Aliteus* Candèze, 1857

*Aliteus* Candèze, 1857: 197. Gender: masculine. Type species: *Alausreichei* Candèze, 1857: 197, by subsequent designation (Hyslop 1921: 625).

####### Genus *Aphileus* Candèze, 1857

*Aphileus* Candèze, 1857: 184. Gender: masculine. Type species: *Aphileuslucanoides* Candèze, 1857: 184, by subsequent designation (Hyslop 1921: 628).

*Dorcostoma* Newman, 1857: 52. Gender: neuter. Type species: *Elaterjansoni* Newman, 1857: 52, by monotypy. Synonymised with *Aphileus* Candèze, 1857 by Harold (1869: 1496).

*Ophileus*: Desmarest 1860: pl. 16 [unavailable name, incorrect subsequent spelling not in prevailing usage].

####### Genus *Austrocalais* Neboiss, 1967

*Austrocalais* Neboiss, 1967: 261. Gender: masculine. Type species: *Austrocalaispogonodes* Neboiss, 1967: 261, by original designation.

####### Genus *Calais* Laporte, 1838

*Calais* Laporte, 1838: 9. Gender: masculine. Type species: *Calaissenegalensis* Laporte, 1838: 9 (syn. of *Elaterexcavatus* Fabricius, 1801: 230), by subsequent designation (Hyslop 1921: 632). The names *Calais*Rafinesque (1815: 99) [Crustacea] and *Calais*Boisduval (1836: 584) [Lepidoptera; see Hemming (1967)] are unavailable.

####### Genus *Catelanus* Fleutiaux, 1942

*Catelanus* Fleutiaux, 1942: 112. Gender: masculine. Type species: *Hemirhipustrilineatus* Laporte, 1838: 12, by monotypy.

*Catelaus*: Schimmel and Tarnawski 2012: 16 [unavailable name, incorrect subsequent spelling not in prevailing usage].

####### Genus *Chalcolepidinus* Pjatakowa, 1941

*Chalcolepidinus* Pjatakowa, 1941: 104. Gender: masculine. Type species: *Chalcolepidinusalbopilosus* Pjatakowa, 1941: 105, by original designation.

####### Genus *Chalcolepidius* Eschscholtz, 1829

*Chalcolepidius* Eschscholtz, 1829: 32. Gender: masculine. Type species: *Elatersulcatus* Fabricius, 1777: 234, by subsequent designation (Duponchel 1843a: 371).

*Charmionus* Gistel, 1834: 11. Gender: masculine. Type species: *Elaterporcatus* Linnaeus, 1767: 652, by subsequent designation (Bousquet and Bouchard 2017: 124). Synonymised with *Chalcolepidius* Eschscholtz, 1829 by Bousquet and Bouchard (2017: 124).

*Hypomochlius* Gistel, 1848b: 128. Gender: masculine. Unnecessary replacement name for *Chalcolepidius* Eschscholtz, 1829.

*Chalcolepidus*: Woodworth 1913: 197 [unavailable name, incorrect subsequent spelling not in prevailing usage].

####### Genus *Chalcolepis* Candèze, 1857

*Chalcolepis* Candèze, 1857: 244. Gender: feminine. Type species: *Chalcolepisluczotii* Candèze, 1857: 245, by monotypy.

*Chalcopedis*: Desmarest 1860: 25 [unavailable name, incorrect subsequent spelling not in prevailing usage].

*Chacolepis*: Vats and Kashyap 1992: 193 [unavailable name, incorrect subsequent spelling not in prevailing usage].

####### Genus *Conobajulus* Van Zwaluwenburg, 1940

*Conobajulus* Van Zwaluwenburg, 1940: 95. Gender: masculine. Type species: *Conobajulusugiensis* Van Zwaluwenburg, 1940: 96, by original designation.

####### Genus *Coryleus* Fleutiaux, 1942

*Coryleus* Fleutiaux, 1942: 94. Gender: masculine. Type species: *Coryleusdesruisseauxi* Fleutiaux, 1942: 94, by monotypy.

####### Genus *Cryptalaus* Ôhira, 1967

*Cryptalaus* Ôhira, 1967b: 97. Gender: masculine. Type species: *Alausputridus* Candèze, 1857: 233, by original designation.

*Paracalais* Neboiss, 1967: 261. Gender: masculine. Type species: *Alaussuboculatus* Candèze, 1857: 229, by original designation. Synonymised with *Cryptalaus* Ôhira, 1967 by Ôhira (1990: 21).

*Clyptalaus*: Ôhira 1990: 20 [unavailable name, incorrect subsequent spelling not in prevailing usage].

*Cryotalaus*: Ôhira 1993: 33 [unavailable name, incorrect subsequent spelling not in prevailing usage].

*Crytalaus*: Jiang 1993: 26 [unavailable name, incorrect subsequent spelling not in prevailing usage].

*Paracalis*: Casari and Costa 1998: 703 [unavailable name, incorrect subsequent spelling not in prevailing usage].

####### Genus *Euphemus* Laporte, 1838

*Euphemus* Laporte, 1838: 7. Gender: masculine. Type species: *Elaterfasciatus* Drury, 1782: pl. XLVIII (syn. of *Elaterquadrimaculatus* Olivier, 1790: 20), by monotypy. The name *Euphemus* Rafinesque, 1815: 144 [Mollusca; see Bieler and Petit 2011] is unavailable.

*Eleuphemus* Hyslop, 1921: 644. Gender: masculine. Unnecessary replacement name for *Euphemus* Laporte, 1838.

####### Genus *Fusimorphus* Fleutiaux, 1942

*Fusimorphus* Fleutiaux, 1942: 116. Gender: masculine. Type species: *Hemirhipussubmetallicus* Fleutiaux, 1924: 184, by monotypy.

####### Genus *Hemirhipus* Berthold, 1827

*Hemirhipus* Berthold, 1827: 336. Gender: masculine. Type species: *Elaterlineatus* Olivier, 1790: 10, by monotypy.

*Hemirrhipus*: Laporte 1838: 12 [unavailable name, incorrect subsequent spelling not in prevailing usage].

*Hemirhipes*: Desmarest 1860: pl. 14 [unavailable name, incorrect subsequent spelling not in prevailing usage].

*Hemirhipis*: Horn 1886: 137 [unavailable name, incorrect subsequent spelling not in prevailing usage].

*Hemirrhypus*: Guérin 1953: 156 [unavailable name, incorrect subsequent spelling not in prevailing usage].

####### Genus *Lycoreus* Candèze, 1857

*Iphis* Laporte, 1838: 7. Gender: feminine. Type species: *Iphisglauca* Laporte, 1838: 8, by subsequent designation (Chevrolat 1845: 106). Preoccupied by *Iphis* Leach, 1817: 25 [Crustacea: Malacostraca: Decapoda].

*Lucarius* Gistel, 1848b: ix. Gender: masculine. Replacement name for *Iphis* Laporte, 1838. Gistel (1848b: xi) also proposed *Lucarius* as a replacement name for *Ocypus* Leach, 1819 (Staphylinidae) in the same work. We act as First Revisers (ICZN 1999, Art. 24.2) and give precedence to *Lucarius* Gistel, 1848a: xi in Staphylinidae and therefore *Lucarius* Gistel, 1848b: ix in Elateridae is treated as a junior homonym.

*Lycoreus* Candèze, 1857: 206. Gender: masculine. Replacement name for *Iphis* Laporte, 1838. This genus has been treated as valid with *Lycoreusregalis* Candèze, 1857: 209 as its type species by subsequent designation by Hyslop (1921: 654). Candèze (1857: 200, 207) however clearly stated that *Lycoreus* was proposed as a replacement name for the preoccupied name *Iphis* Laporte, 1838 and therefore *Lycoreus* has the same type species.

*Abiphis* Fleutiaux, 1926: 92. Gender: feminine. Replacement name for *Iphis* Laporte, 1838. This genus has been treated as valid with *Elaternobilis* Illiger, 1800: 116 as its type species by original designation. Fleutiaux (1926: 92), however, clearly stated that *Abiphis* was proposed as a replacement name for the preoccupied name *Iphis* Laporte, 1838 and therefore *Abiphis* has the same type species.

*Lacais* Fleutiaux, 1942: 109. Gender: feminine. Type species: *Iphisglauca* Laporte, 1838: 8, by monotypy. Junior objective synonym of *Iphis* Laporte, 1838 and of the replacement names proposed for *Iphis*.

####### Genus *Mocquerysia* Fleutiaux, 1899

*Mocquerysia* Fleutiaux, 1899: 369. Gender: feminine. Type species: *Mocquerysiabicolor* Fleutiaux, 1899: 369, by subsequent designation (Hyslop 1921: 657).

*Moquerisia*: Dolin 2000: 19 [unavailable name, incorrect subsequent spelling not in prevailing usage].

####### Genus *Neoabiphis* Kundrata & Bouchard, gen. n.

Type species: *Elaternobilis* Illiger, 1800: 116, **herein designated**. Gender: feminine. Genus *Abiphis* Fleutiaux, 1926 has been treated as valid with *Elaternobilis* Illiger, 1800 as its type species by original designation. However, Fleutiaux (1926: 92) clearly stated that *Abiphis* was proposed as a replacement name for the preoccupied name *Iphis* Laporte, 1838 and therefore *Abiphis* has the same type species. Because the name *Abiphis* is a synonym of genus *Lycoreus* Candèze, 1857 (= replacement name for *Iphis* Laporte, 1838), we establish here a new genus based on *Elaternobilis* Illiger, 1800, which was used as type species for *Abiphis*. See Casari (2008: 165–166, couplet 12 of her key) for diagnostic character states (ICZN 1999, Art. 13.1.2). All species hitherto classified under *Abiphis* Fleutiaux, 1926 (see Casari-Chen 1994: 191) are here transferred to *Neoabiphis* Kundrata & Bouchard, gen. n., and the following new combinations are proposed: *N.candezei* (Alluaud, 1896), comb. n., *N.fairmairei* (Fleutiaux, 1903), comb. n., *N.goudoti* (Fleutiaux, 1942), comb. n., *N.insignis* (Klug, 1833), comb. n., *N.nobilis* (Illiger, 1800), comb. n., and *N.viettei* (Girard, 1966), comb. n.

####### Genus *Neocalais* Girard, 1971

*Neocalais* Girard, 1971: 587. Gender: masculine. Type species: *Alausmacer* Candèze, 1878a: lv, by original designation.

####### Genus *Neolycoreus* Kundrata & Bouchard, gen. n.

Type species: *Lycoreusregalis* Candèze, 1857: 209, **herein designated**. Gender: masculine. Genus *Lycoreus* Candèze, 1857 has been treated as valid with *Lycoreusregalis* Candèze, 1857 as its type species by subsequent designation. However, Candèze (1857: 200, 207) clearly stated that *Lycoreus* was proposed as a replacement name for the preoccupied name *Iphis* Laporte, 1838 and therefore *Lycoreus* has the same type species. Because the name *Lycoreus* is now treated as a valid replacement name for *Iphis* Laporte, 1838, we establish here a new genus based on *Lycoreusregalis* Candèze, 1857, which was used as type species for *Lycoreus*. See Casari (2008: 166, couplet 15 of her key) for diagnostic character states (ICZN 1999, Art. 13.1.2). Subsequent designation of type species for *Lycoreus* (i.e., *Iphistriocellata* Laporte, 1838) by Fleutiaux (1942: 95) was invalid. All species hitherto classified under *Lycoreus* Candèze, 1857 (see Casari-Chen 1994: 185) are here transferred to *Neolycoreus* Kundrata & Bouchard, gen. n., and the following new combinations are proposed: *N.alluaudi* (Candèze, 1900), comb. n., *N.corpulentus* (Candèze, 1899), comb. n., *N.cyclops* (Candèze, 1865), comb. n., *N.decorsei* (Fleutiaux, 1903), comb. n., *N.dux* (Candèze, 1857), comb. n., *N.goudotii* (Laporte, 1838), comb. n., *N.madagascariensis* (Gory, 1832), comb. n., *N.oculipennis* (Fairmaire, 1903), comb. n., *N.orbiculatus* (Schwarz, 1901), comb. n., *N.regalis* (Candèze, 1857), comb. n., *N.sicardi* (Fleutiaux, 1942), comb. n., *N.triangularis* (Fleutiaux, 1942), comb. n., *N.triocellatus* (Laporte, 1838), comb. n., and *N.vicinus* (Fleutiaux, 1942), comb. n.

####### Genus *Nycterilampus* Montrouzier, 1860

*Nycterilampus* Montrouzier, 1860: 258. Gender: masculine. Type species: *Nycterilampuslifuanus* Montrouzier, 1860: 258, by monotypy.

*Nycterolampus*: Fleutiaux 1891: 391 [unavailable name, incorrect subsequent spelling not in prevailing usage]. We follow Costa et al. (2009: 48) in treating this name as an incorrect subsequent spelling of *Nycterilampus* Montrouzier, 1860.

####### Genus *Pherhimius* Fleutiaux, 1942

*Pherhimius* Fleutiaux, 1942: 114. Gender: masculine. Type species: *Elaterfascicularis* Fabricius, 1787: 171, by original designation.

####### Genus *Phertetrigus* Schimmel & Tarnawski, 2012

*Phertetrigus* Schimmel & Tarnawski, 2012: 223. Gender: masculine. Type species: *Tetrigusparryi* Candèze, 1865: 18, by original designation.

####### Genus *Phibisa* Fleutiaux, 1942

*Phibisa* Fleutiaux, 1942: 105. Gender: feminine. Type species: *Ctenicerapupieri* Fleutiaux, 1903: 228, by original designation.

####### Genus *Propalaus* Casari, 2008

*Propalaus* Casari, 2008: 164. Gender: masculine. Type species: *Chalcolepidiusharoldi* Candèze, 1878a: lv, by original designation.

####### Genus *Pseudocalais* Girard, 1971

*Pseudocalais* Girard, 1971: 588. Gender: masculine. Type species: *Alauslongipennis* Schwarz, 1900: 145, by original designation.

####### Genus *Punctodensus* Patwardhan, Schimmel & Athalye, 2009

*Punctodensus* Patwardhan, Schimmel & Athalye, 2009: 54. Gender: masculine. Type species: *Punctodensusindicus* Patwardhan, Schimmel & Athalye, 2009: 55, by original designation.

####### Genus *Saltamartinus* Casari, 1996

*Saltamartinus* Casari, 1996: 386. Gender: masculine. Type species: *Hemirhipusdecorus*Candèze 1857: 254, by original designation.

####### Genus *Thoramus* Sharp, 1877

*Thoramus* Sharp, 1877: 399. Gender: masculine. Type species: *Thoramuswakefieldi* Sharp, 1877: 399, by subsequent designation (Hyslop 1921: 672).

###### Subtribe Ludioctenina Jakobson, 1913

Ludioctenina Jakobson, 1913: 755. Type genus: *Ludioctenus* Fairmaire, 1893.

Tetrigusina Schimmel & Tarnawski, 2012: 116. Type genus: *Tetrigus* Candèze, 1857. The correct stem based on genus *Tetrigus* is Tetrig-, however Schimmel and Tarnawski (2012) used the whole genus name as stem (as required by ICZN 1999, Art. 29.6) in order to avoid homonymy with Tetrigidae Rambur, 1838: 64, previously established in the order Orthoptera.

####### Genus *Collisarius* Schimmel & Tarnawski, 2012

*Collisarius* Schimmel & Tarnawski, 2012: 118. Gender: masculine. Type species: *Tetrigusbakeri* Fleutiaux, 1941a: 18, by original designation.

####### Genus *Cuneateus* Schimmel & Tarnawski, 2012

*Cuneateus* Schimmel & Tarnawski, 2012: 118. Gender: masculine. Type species: *Cuneateusjingkei* Schimmel & Tarnawski, 2012: 144, by original designation.

####### Genus *Ludioctenus* Fairmaire, 1893

*Ludioctenus* Fairmaire, 1893: lxviii. Gender: masculine. Type species: *Ludioctenusakbesianus* Fairmaire, 1893: lxix (syn. of *Tetriguscyprius* Baudi di Selve, 1871: 50), by monotypy. Previously considered as a synonym of *Tetrigus* Candèze, 1857 but removed from synonymy by Schimmel & Tarnawski (2012: 117).

*Elatrigus* Reitter, 1905: 10. Gender: masculine. Type species: *Tetriguscyprius* Baudi di Selve, 1871: 50, by monotypy. Synonymised with *Ludioctenus* Fairmaire, 1893 by Winkler & Buysson in Winkler (1925: 580).

####### Genus *Platianellus* Schimmel & Tarnawski, 2012

*Platianellus* Schimmel & Tarnawski, 2012: 116. Gender: masculine. Type species: *Platianelluscasariae* Schimmel & Tarnawski, 2012: 165, by original designation.

####### Genus *Tetrigus* Candèze, 1857

*Tetrigus* Candèze, 1857: 254. Gender: masculine. Type species: *Tetrigusparallelus* Candèze, 1857: 255, by subsequent designation (Hyslop 1921: 671).

##### Tribe Oophorini Gistel, 1848

Oophoridae Gistel, 1848a: [5]. Type genus: *Oophorus* Dejean, 1833 (syn. of *Aeolus* Eschscholtz, 1829).

Monocrépidiites Candèze, 1859: 176. Type genus: *Monocrepidius* Eschscholtz, 1829 (syn. of *Conoderus* Eschscholtz, 1829).

Drasteriini Houlbert, 1912: 184. Type genus: *Drasterius* Eschscholtz, 1829. Not Drasteriini Wiltshire, 1976: 160 (Lepidoptera); see Bouchard et al. (2011).

Aeolina: Jakobson 1913: 747. Type genus: *Aeolus* Eschscholtz, 1829. Unavailable family-group name; see Bouchard et al. (2011).

Conoderinae Fleutiaux, 1919: 58. Type genus: *Conoderus* Eschscholtz, 1829. Not Conoderinae Schönherr, 1833: 26 (Coleoptera: Curculionidae); see Bouchard et al. (2011).

Pachyderinae Fleutiaux, 1919: 57. Type genus: *Pachyderes* Guérin-Méneville, 1829.

Monocrepidina: Leng 1920: 167 [unavailable name, incorrect subsequent spelling not in prevailing usage].

Canoderinae: Miwa 1927: 12 [unavailable name, incorrect subsequent spelling not in prevailing usage].

Monocorepidiinae: Mouchet 1950: 186 [unavailable name, incorrect subsequent spelling not in prevailing usage].

###### Genus *Aeoloderma* Fleutiaux, 1928

*Aeoloderma* Fleutiaux, 1928a: 135. Gender: neuter. Type species: *Elatercrucifer* Rossi, 1790: 183, by original designation.

*Aeloderma*: Fleutiaux 1928a: 135 [unavailable name, incorrect original spelling not in prevailing usage (ICZN 1999, Art. 19.3)]; First Reviser (Art. 24.2): Fleutiaux (1930: 273).

*Aeleoderma*: Fleutiaux 1932a: 15 [unavailable name, incorrect subsequent spelling not in prevailing usage].

*Aeolocerma*: Miwa 1934: 243 [unavailable name, incorrect subsequent spelling not in prevailing usage].

*Atoloderma*: Fleutiaux 1934a: 182 [unavailable name, incorrect subsequent spelling not in prevailing usage].

*Aoloderma*: Platia and Marini 1990: 32 [unavailable name, incorrect subsequent spelling not in prevailing usage].

###### Genus *Aeoloides* Schwarz, 1906

*Aeoloides* Schwarz, 1906a: 109. Gender: masculine. Type species: *Heteroderessequester* Candèze, 1859: 378, by subsequent designation (Hyslop 1921: 624).

###### Genus *Aeolosomus* Dolin, 1982

*Aeolosomus* Dolin, 1982: 106. Gender: masculine. Type species: *Cryptohypnusrossii* Germar, 1844: 148, original designation.

*Aolosomus*: Platia and Marini 1990: 33 [unavailable name, incorrect subsequent spelling not in prevailing usage].

###### Genus *Aeolus* Eschscholtz, 1829

*Aeolus* Eschscholtz, 1829: 33. Gender: masculine. Type species: *Elaterscriptus* Fabricius, 1801: 244, by subsequent designation (Lacordaire 1857: 187).

*Oophorus* Dejean, 1833: 93. Gender: masculine. Type species: *Elaterelegans* Fabricius, 1792: 230, by subsequent designation (Hyslop 1921: 659). Synonymised with *Aeolus* Eschscholtz, 1829 by Cate et al. (2007: 104).

*Trypheus* Gistel, 1834: 12. Gender: masculine. Type species: *Elaterelegans* Fabricius, 1792: 230, by monotypy. Junior objective synonym of *Oophorus* Dejean, 1833 (also see Bousquet and Bouchard 2017: 125).

*Aelus*: Chagnon 1935: 133 [unavailable name, incorrect subsequent spelling not in prevailing usage].

*Aiolus*: Wilson 1941: 26 [unavailable name, incorrect subsequent spelling not in prevailing usage].

*Aeolous*: Dolin 1978: 4 [unavailable name, incorrect subsequent spelling not in prevailing usage].

###### Genus *Apochresis* Candèze, 1882

*Apochresis* Candèze, 1882: 46. Gender: feminine. Type species: *Apochresisaspera* Candèze, 1882: 46, by monotypy [the spelling *asper* is an incorrect original spelling].

###### Genus *Babadrasterius* Ôhira, 1994

*Babadrasterius* Ôhira, 1994: 224. Gender: masculine. Type species: *Babadrasteriusurabensis* Ôhira, 1994: 224, by original designation.

###### Genus *Deronocus* Johnson, 1997

*Deroconus* Johnson, 1995: 60. Gender: masculine. Type species: *Ctenicerasleeperi* Becker, 1973: 1529, by original designation. Preoccupied by *Deroconus* Jekel, 1854: 9bis [Coleoptera: Curculionidae].

*Deronocus* Johnson, 1997: 284. Gender: masculine. Replacement name for *Deroconus* Johnson, 1995.

###### Genus *Drasterius* Eschscholtz, 1829

*Drasterius* Eschscholtz, 1829: 33. Gender: masculine. Type species: *Elaterbimaculatus* Rossi, 1790: 182, by subsequent designation (Curtis 1838: 694), see Sánchez-Ruiz (1996: 48).

*Dresterius*: Brullé 1832: 141 [unavailable name, incorrect subsequent spelling not in prevailing usage].

*Epistrophus* Gistel, 1834: 12. Gender: masculine. Type species: *Elaterbimaculatus* Rossi, 1790: 182, by monotypy. Junior objective synonym of *Drasterius* Eschscholtz, 1829 (also see Bousquet and Bouchard 2017: 126).

*Prodrasterius* Fleutiaux, 1927b: 91. Gender: masculine. Type species: *Drasteriusbrahminus* Candèze, 1859: 426, by monotypy. Synonymised with *Drasterius* Eschscholtz, 1829 by Platia & Schimmel (1997: 300).

*Draesterius*: Leseigneur 1955: 86 [unavailable name, incorrect subsequent spelling not in prevailing usage].

*Prodratsreius*: Schimmel and Tarnawski 2012: 12 [unavailable name, incorrect subsequent spelling not in prevailing usage].

###### Genus *Gahanus* Platia, 2012

*Gahanus* Platia, 2012: 132. Gender: masculine. Type species: *Gahanussocotranus* Platia, 2012: 133, by original designation.

###### Genus *Grammephorus* Solier, 1851

*Grammephorus* Solier, 1851: 20. Gender: masculine. Type species: *Grammephorusrufipennis* Solier, 1851: 21, by monotypy.

*Grammophorus*: Candèze 1859: 417 [unavailable name, incorrect subsequent spelling not in prevailing usage].

###### Genus *Hartenius* Platia, 2007

*Hartenius* Platia, 2007: 200. Gender: masculine. Type species: *Harteniusnarci* Platia, 2007: 200, by original designation.

###### Genus *Heteroderes* Latreille, 1834

*Heteroderes* Latreille, 1834: 155. Gender: masculine. Type species: *Heteroderesfuscus* Latreille, 1834: 155, by monotypy.

*Exaeolus* Gozis, 1886: 22. Gender: masculine. Type species: *Oophorusalgirinus* Lucas, 1847: 166, by original designation. Synonymised with *Heteroderes* Latreille, 1834 by Schenkling (1925: 126). Górriz (1902: 180) and Schwarz (1906a: 106) had treated the type species of *Exaeolus* Gozis, 1886 as belonging under *Heteroderes* Latreille, 1834 without formally proposing the synonymy of *Exaeolus* with *Heteroderes*.

*Heterodes*: Sánchez-Ruiz & Sáez-Bolaño, 1994: 121 [unavailable name, incorrect subsequent spelling not in prevailing usage].

###### Genus *Melanthoides* Candèze, 1865

*Melanthoides* Candèze, 1865: 23. Gender: masculine. Type species: *Melanthoideslatimanus* Candèze, 1865: 24, by monotypy.

*Malanthoides*: Dolin 1975: 1626 [unavailable name, incorrect subsequent spelling not in prevailing usage].

###### Genus *Monocrepidius* Eschscholtz, 1829

*Conoderus* Eschscholtz, 1829: 31. Gender: masculine. Type species: *Conoderusfuscofasciatus* Eschscholtz, 1829: 31, by subsequent designation (Duponchel 1843b: 188).

*Monocrepidius* Eschscholtz, 1829: 31. Gender: masculine. Type species: *Monocrepidiuspallipes* Eschscholtz, 1829: 32, by subsequent designation (Hyslop 1921: 657). First Reviser (Art. 24.2) found (*Monocrepidius* Eschscholtz, 1829 vs. *Conoderus* Eschscholtz, 1829) is Germar (1839: 223); see Sánchez-Ruiz (1996: 184).

*Monocepidius*: Henshaw 1885: 67 [unavailable name, incorrect subsequent spelling not in prevailing usage].

*Silene* Broun, 1893: 1135. Gender: feminine. Type species: *Silenebrunnea* Broun, 1893: 1136, by monotypy. Synonymised with *Conoderus* Eschscholtz, 1829 by Calder (1996: 79).

*Monocrepedius*: Schaeffer 1909: 436 [unavailable name, incorrect subsequent spelling not in prevailing usage].

*Monocrepidus*: Wolcott 1923: 86 [unavailable name, incorrect subsequent spelling not in prevailing usage].

*Conoderes*: Van Dyke, 1932: 294 [unavailable name, incorrect subsequent spelling not in prevailing usage].

###### Genus *Nanseia* Kishii, 1985

*Nanseia* Kishii, 1985: 5. Gender: feminine. Type species: *Prodrasteriuserabuensis* Kishii, 1966: 8, by monotypy.

###### Genus *Neodrasterius* Kishii, 1996

*Neodrasterius* Kishii, 1996: 90. Gender: masculine. Type species: *Neodrasteriuskiyoyamai* Kishii, 1996: 91, by original designation.

###### Genus *Nipponodrasterius* Kishii, 1966

*Nipponodrasterius* Kishii, 1966: 9. Gender: masculine. Type species: *Nipponodrasteriusalpicola* Kishii, 1966: 9, by original designation.

###### Genus *Pachyderes* Guérin-Méneville, 1829

*Pachyderes* Guérin-Méneville, 1829: plate 12. Gender: masculine. Type species: *Pachyderesruficollis* Guérin-Méneville, 1829: plate 12, by monotypy.

###### Genus *Paraheteroderes* Girard, 2017

*Paraheteroderes* Girard, 2017: 71. Gender: masculine. Type species: *Heterodereskordofanus* Candèze, 1859: 364, by original designation.

###### Genus *Phedomenus* Candèze, 1889

*Phedomenus* Candèze, 1889: 89. Gender: masculine. Type species: *Phedomenusvenustus* Candèze, 1889: 90, by subsequent designation (Hyslop 1921: 664).

####### Subgenus Domenephus Fleutiaux, 1932

*Domenephus* Fleutiaux, 1932c: 277. Gender: masculine. Type species: *Diploconusflavangulus* Candèze, 1895: 59, by original designation.

####### Subgenus Phedomenus Candèze, 1889

*Phedomenus* Candèze, 1889: 89. Gender: masculine. Type species: *Phedomenusvenustus* Candèze, 1889: 90, by subsequent designation (Hyslop 1921: 664).

###### Genus *Pseudaeolus* Candèze, 1891

*Pseudaeolus* Candèze, 1891: 77. Gender: masculine. Type species: *Aeolusaustralis* Candèze, 1859: 284, by subsequent designation (Hyslop 1921: 667).

###### Genus *Telesus* Candèze, 1880

*Telesus* Candèze, 1880: 9. Gender: masculine. Type species: *Telesusritsemae* Candèze, 1880: 10, by monotypy.

##### Tribe Platycrepidiini Costa & Casari-Chen, 1993

Eudactylites Candèze, 1859: 153. Type genus: *Eudactylus* Sallé, 1855. Preoccupied genus name, not *Eudactylus* Fitzinger, 1843 [Reptilia]; syn. of *Platycrepidius* Candèze, 1859. Permanently invalid, based on preoccupied type genus; see Bouchard et al. (2011).

Platycrepidiini Costa & Casari-Chen, 1993: 62. Type genus: *Platycrepidius* Candèze, 1859.

###### Genus *Platycrepidius* Candèze, 1859

*Eudactylus* Sallé, 1855: 266. Gender: masculine. Type species: *Eudactyluswapleri* Sallé, 1855: 267, by monotypy. Preoccupied by *Eudactylus* Fitzinger, 1843: 67 (Reptilia).

*Platycrepidius* Candèze, 1859: 159. Gender: masculine. Type species: *Eudactyluswapleri* Sallé, 1855: 267, by subsequent designation (Hyslop 1921: 665). Originally proposed as a synonym of *Eudactylus* Sallé, 1855 and subsequently treated as valid (ICZN 1999, Art. 11.6). Objective synonym of *Eudactylus* Sallé, 1855.

*Platicrepidius*: Motschulsky 1859: 358 [unavailable name, incorrect subsequent spelling not in prevailing usage; see Hyslop (1921: 665)].

*Tyleudacus* Fleutiaux, 1922: 429. Gender: masculine. Unnecessary replacement name for *Eudactylus* Sallé, 1855.

##### Tribe Pseudomelanactini Arnett, 1967

Pseudomelanactini Arnett, 1967: 111. Type genus: *Pseudomelanactes* Mathieu, 1961 [synonym of *Anthracalaus* Fairmaire, 1888].

Pseudomelactini: Casari 2008: 141 [unavailable name, incorrect subsequent spelling not in prevailing usage].

###### Genus *Anthracalaus* Fairmaire, 1888

*Anthracalaus* Fairmaire, 1888: 349. Gender: masculine. Type species: *Alauswestermanni* Candèze, 1857: 216, by subsequent designation (Hyslop 1921: 628).

*Anthracralaus*: Fleutiaux (1927a: 101) [unavailable name, incorrect subsequent spelling not in prevailing usage].

*Pseudomelanactes* Mathieu, 1961: 474. Gender: masculine. Type species: *Melanactesagrypnoides* Van Dyke, 1932: 446, by original designation. Synonymised with *Anthracalaus* Fairmaire, 1888 by Calder & Hayek (1992: 17).

###### Genus *Lanelater* Arnett, 1952

*Amaurus* Laporte, 1840: 237. Gender: masculine. Type species: *Amaurussenegalensis* Laporte, 1840: 237 (syn. of *Elaternotodonta* Latreille, 1827: 275), by subsequent designation (Hyslop 1921: 625). Preoccupied by *Amaurus* Burmeister, 1834: 294 [Hemiptera]. Synonymised with *Lanelater* Arnett, 1952 by Arnett (1952: 105).

*Lanelater* Arnett, 1952: 105. Gender: masculine. Type species: *Agrypnusschotti* LeConte, 1853: 492, by original designation.

##### Tribe Pyrophorini Candèze, 1863

Pyrophorites Candèze, 1863: 3. Type genus: *Pyrophorus* Billberg, 1820.

###### Subtribe Hapsodrilina Costa, 1975

Hapsodrilina Costa, 1975: 88. Type genus: *Hapsodrilus* Costa, 1975.

####### Genus *Hapsodrilus* Costa, 1975

*Hapsodrilus* Costa, 1975: 90. Gender: masculine. Type species: *Pyrophorusignifer* Germar, 1841: 46, by original designation.

####### Genus *Ptesimopsia* Costa, 1975

*Ptesimopsia* Costa, 1975: 91. Gender: feminine. Type species: *Pyrophoruscandezei* Fauvel, 1860: 307, by original designation.

####### Genus *Pyroptesis* Costa, 1975

*Pyroptesis* Costa, 1975: 90. Gender: feminine. Type species: *Pyrophoruscincticollis* Germar, 1841: 44, by original designation.

####### Genus *Sooporanga* Costa, 1975

*Sooporanga* Costa, 1975: 89. Gender: masculine. Type species: *Pyrophorusformosus* Germar, 1841: 41, by original designation.

###### Subtribe Nyctophyxina Costa, 1975

Nyctophyxina Costa, 1975: 85. Type genus: *Nyctophyxis* Costa, 1975 [incorrect stem formation in prevailing usage; see Bouchard et al. (2011)].

####### Genus *Cryptolampros* Costa, 1975

*Cryptolampros* Costa, 1975: 88. Gender: masculine. Type species: *Pyrophoruscoecus* Germar, 1841: 40, by original designation.

####### Genus *Noxlumenes* Costa, 1975

*Noxlumenes* Costa, 1975: 87. Gender: masculine (based on the ICZN 1999, Art. 30.2.4; the specific name *bardus* is a noun in apposition; Art. 31.2.2). Type species: *Noxlumenesbardus* Costa, 1975: 87, by original designation.

####### Genus *Nyctophyxis* Costa, 1975

*Nyctophyxis* Costa, 1975: 86. Gender: feminine (erroneously treated as masculine in original description; the Greek noun φύξις (latinised phyxis, meaning refuge) is present in the Greek dictionaries with the feminine gender; ICZN 1999, Art. 30.1.2). Type species: *Pyrophorusocellatus* Germar, 1841: 49, by original designation.

*Nyctophixis*: Costa 1975: 86 [incorrect original spelling (ICZN 1999, Art. 19.3; First Revisers (Art. 24.2): Sáiz et al. (1990: 70)].

*Nyctophysis*: Bouchard et al. (2011: 310) [unavailable name, incorrect subsequent spelling not in prevailing usage].

###### Subtribe Pyrophorina Candèze, 1863

Pyrophorites Candèze, 1863: 3. Type genus: *Pyrophorus* Billberg, 1820.

####### Genus *Deilelater* Costa, 1975

*Deilelater* Costa, 1975: 108. Gender: masculine. Type species: *Pyrophorusphysoderus* Germar, 1841: 36, by original designation.

####### Genus *Fulgeochlizus* Costa, 1975

*Fulgeochlizus* Costa, 1975: 103. Gender: masculine. Type species: *Pyrophorusbruchi* Candèze, 1897: 66, by original designation.

####### Genus *Hifo* Candèze, 1882

*Hifo* Candèze, 1882: 94. Gender: masculine. Type species: *Hifopacificus* Candèze, 1882: 94, by monotypy.

####### Genus *Hifoides* Schwarz, 1906

*Hifoides* Schwarz, 1906b: 154. Gender: masculine. Type species: *Pyrophorussemiotoides* Schwarz, 1906b: 154, by monotypy.

####### Genus *Hypsiophthalmus* Latreille, 1834

*Hypsiophthalmus* Latreille, 1834: 145. Gender: masculine. Type species: *Pyrophorusbuphthalmus* Eschscholtz, 1829: 32, by monotypy (see Costa 1975: 97).

*Belania* Laporte, 1840: 236. Gender: feminine. Type species: *Pyrophorusbuphthalmus* Eschscholtz, 1829: 32, by monotypy. Junior objective synonym of *Hypsiophthalmus* Latreille, 1834.

####### Genus *Ignelater* Costa, 1975

*Stilpnus* Laporte, 1840: 236. Gender: masculine. Type species: *Pyrophorushavaniensis* Laporte, 1840: 236, by subsequent designation (Hyslop 1921: 671). Preoccupied by *Stilpnus* Gravenhorst, 1829: 664 [Hymenoptera].

*Ignelater* Costa, 1975: 105. Gender: masculine. Replacement name for *Stilpnus* Laporte, 1840.

####### Genus *Lygelater* Costa, 1975

*Lygelater* Costa, 1975: 106. Gender: masculine. Type species: *Pyrophorusfulgidus* Germar, 1841: 24, by original designation.

####### Genus *Metapyrophorus* Rosa & Costa, 2009

*Metapyrophorus* Rosa & Costa, 2009: 45. Gender: masculine. Type species: *Metapyrophoruspharolim* Rosa & Costa, 2009: 45, by monotypy.

####### Genus *Opselater* Costa, 1975

*Opselater* Costa, 1975: 103. Gender: masculine. Type species: *Elaterpyrophanus* Illiger, 1807: 149, by original designation.

####### Genus *Phanophorus* Solier, 1851

*Phanophorus* Solier, 1851: 26. Gender: masculine. Type species: *Phanophorusparallelus* Solier, 1851: 27, by subsequent designation (Hyslop 1921: 663).

*Phamophorus*: Schwarz 1906a: 210 [unavailable name, incorrect subsequent spelling not in prevailing usage].

*Phantophorus*: Schimmel and Taranwski 2012: 114 [unavailable name, incorrect subsequent spelling not in prevailing usage].

####### Genus *Photophorus* Candèze, 1863

*Photophorus* Candèze, 1863: 72. Gender: masculine. Type species: *Photophorusjansonii* Candèze, 1863: 73, by subsequent designation (Hyslop 1921: 664).

####### Genus *Pyrearinus* Costa, 1975

*Pyrearinus* Costa, 1975: 99. Gender: masculine. Type species: *Pyrophorusnyctolampis* Germar, 1841: 54, by original designation.

####### Genus *Pyrophorus* Billberg, 1820

*Phosphorus*: Voet 1806: Tab. 43, fig. 17 [unavailable name, work not consistently binominal (ICZN 1999, Art. 11.4)]. We follow other authors (e.g., Alonso-Zarazaga and Lyal 1999: 8) in considering the names in Voet’s publication not consistently binominal. The genus-group name *Phosphorus* has not been made available in Elateridae to our knowledge.

*Pyrophorus* Billberg, 1820: 20. Gender: masculine. Type species: *Elaternoctilucus* Linnaeus, 1758: 404, by subsequent designation (Desmarest 1860: 29).

*Hedonius* Gistel, 1834: 11. Gender: masculine. Type species: *Elaternoctilucus* Linnaeus, 1758: 404, by subsequent designation (Bousquet and Bouchard 2017: 124). Junior objective synonym of *Pyrophorus* Billberg, 1820 (also see Bousquet and Bouchard 2017: 124).

*Phyrophorus*: Wolcott 1936: 211 [unavailable name, incorrect subsequent spelling not in prevailing usage].

####### Genus *Vesperelater* Costa, 1975

*Vesperelater* Costa, 1975: 109. Gender: masculine. Type species: *Pyrophorusornamentum* Germar, 1841: 39, by original designation.

#### Agrypninae *incertae sedis*

##### Genus *Anathesis* Candèze, 1865

*Anathesis* Candèze, 1865: 21. Gender: feminine. Type species: *Anathesislaconoides* Candèze, 1865: 21, by monotypy.

##### Genus *Antitypus* Candèze, 1882

*Antitypus* Candèze, 1882: 70. Gender: masculine. Type species: *Elaterinsignitus* Fairmaire & Germain, 1860: 268, by monotypy.

##### Genus *Chrostus* Candèze, 1878

*Chrostus* Candèze, 1878b: clxix. Gender: masculine. Type species: *Chrostusquadrifoveolatus* Candèze, 1878b: clxx, by monotypy.

##### Genus *Dorygonus* Candèze, 1859

*Dorygonus* Candèze, 1859: 182. Gender: masculine. Type species: *Dorygonusamaurus* Candèze, 1859: 186, by subsequent designation (Hyslop 1921: 641).

###### Subgenus Dorygonus Candèze, 1859

*Dorygonus* Candèze, 1859: 182. Gender: masculine. Type species: *Dorygonusamaurus* Candèze, 1859: 186, by subsequent designation (Hyslop 1921: 641).

###### Subgenus Rygodonus Fleutiaux, 1932

*Rygodonus* Fleutiaux, 1932d: 857. Gender: masculine. Type species: *Dorygonusalluaudi* Fleutiaux, 1932d: 863, by original designation.

##### Genus *Macromalocera* Hope, 1834

*Macromalocera* Hope, 1834: 13. Gender: feminine. Type species: *Macromaloceracaenosa* Hope, 1834: 14, by subsequent designation (Hyslop 1921: 654).

#### Subfamily Campyloxeninae Costa, 1975

Campyloxeninae Costa, 1975: 114. Type genus: *Campyloxenus* Fairmaire & Germain, 1860.

##### Genus *Campyloxenus* Fairmaire & Germain, 1860

*Campyloxenus* Fairmaire & Germain, 1860: 6. Gender: masculine. Type species: *Campyloxenuspyrothorax* Fairmaire & Germain, 1860: 6, by monotypy.

##### *Malalcahuello* Arias-Bohart, 2015

*Malalcahuello* Arias-Bohart, 2015: 2. Gender: masculine. Type species: *Malalcahuelloocaresi* Arias-Bohart, 2015: 4, by original designation.

#### Subfamily Hemiopinae Fleutiaux, 1941

Hemiopinae Fleutiaux, 1941b: 31. Type genus: *Hemiops* Laporte, 1838.

Hemiopsinae: Fleutiaux 1947a: 239 [unavailable name, incorrect stem formation (ICZN 1999, Art. 29.3)].

##### Genus *Exoeolus* Broun, 1893

*Exoeolus* Broun, 1893: 1133. Gender: masculine. Type species: *Exoeolusrufescens* Broun, 1893: 1134, by subsequent designation (Hyslop 1921: 646; as *Exaeolus*).

*Exaeolus*: Schwarz 1906a: 161 [unavailable name, incorrect subsequent spelling not in prevailing usage].

*Brounaeolus* Hyslop, 1921: 631. Gender: masculine. Unnecessary replacement name for *Exoeolus* Broun, 1893 (misspelled as *Exaeolus* which is preoccupied by *Exaeolus* Gozis, 1886: 22).

##### Genus *Hemiops* Laporte, 1838

*Oxysternus* Latreille, 1834: 164. Gender: masculine. Type species: *Elatercrassus* Gyllenhal, 1817: 135, by monotypy. Preoccupied by *Oxysternus* Dejean, 1833: 129 [Coleoptera: Histeridae]. Synonymised with *Hemiops* Laporte, 1838 by Candèze (1863: 492).

*Hemiops* Laporte, 1838: 15. Gender: masculine. Type species: *Hemiopsflavus* Laporte, 1838: 15, by subsequent designation (Hyslop 1921: 648).

*Plectrosternus* Lacordaire, 1857: 227. Gender: masculine. Replacement name for *Oxysternus* Latreille, 1834.

*Oxisternus*: Candèze 1863: 492 [unavailable name, incorrect subsequent spelling not in prevailing usage].

*Plectrostenus*: Marschall 1873: 233 [unavailable name, incorrect subsequent spelling not in prevailing usage].

*Plectosternus*: Schwarz 1907: 304 [unavailable name, incorrect subsequent spelling not in prevailing usage].

##### Genus *Legna* Walker, 1858

*Legna* Walker, 1858: 281. Gender: feminine (ICZN 1999, Art. 30.2.1). Type species: *Legnaidonea* Walker, 1858: 281 (= *Plectrosternusrufus* Lacordaire, 1857), by monotypy. Schenkling (1927: 503) used *Plectrosternus* Lacordaire, 1857 as valid with *Legna* Walker, 1858 as a synonym. However *Plectrosternus* Lacordaire, 1857 is a replacement name for *Oxysternus* Latreille, 1834 and therefore it has the same type species as *Oxysternus* (= synonym of the valid name *Hemiops* Laporte, 1838). When *Plectrosternus* Lacordaire, 1857 was moved under *Hemiops* for nomenclatural reasons then this left the genus which previously included the species of *Plectrosternus* sensu Schenkling (1927) (with *Legnaidonea* Walker, 1858 as a synonym of *P.rufus* Lacordaire, 1857) without a valid name. The only available name for that genus is *Legna* Walker, 1858. Therefore, all species hitherto classified under *Plectrosternus*, i.e., *P.rufus* Lacordaire, 1857, *P.convexus* Vats, 1991, *P.coolsi* Schimmel, 1996 and *P.foveatus* Patwardhan & Athalye, 2012, are here transferred to *Legna* Walker, 1858, and new combinations *Legnarufa* (Lacordaire, 1857), comb. n., *L.convexa* (Vats, 1991), comb. n., *L.coolsi* (Schimmel, 1996), comb. n., and *L.foveata* (Patwardhan & Athalye, 2012), comb. n. are proposed.

##### Genus *Parhemiops* Candèze, 1878

*Parhemiops* Candèze, 1878c: cxcviii. Gender: masculine. Type species: *Parhemiopspalliatus* Candèze, 1878c: cxcviii, by monotypy.

*Lincydrus* Fleutiaux, 1932b: 148. Gender: masculine. Type species: *Lincydruscylindricus* Fleutiaux, 1932b: 149, by monotypy. Synonymised with *Parhemiops* Candèze, 1878 by Van Zwaluwenburg (1959: 411).

#### Subfamily Lissominae Laporte, 1835

Lissomidae Laporte, 1835: 178. Type genus: *Lissomus* Dalman, 1824.

##### Tribe Lissomini Laporte, 1835

Lissomidae Laporte, 1835: 178. Type genus: *Lissomus* Dalman, 1824.

Drapetini LeConte, 1863: 44. Type genus: *Drapetes* Dejean, 1821.

Drapetini Dolin, 1975: 1627. Type genus: *Drapetes* Dejean, 1821. Proposed as new without reference to Drapetini LeConte, 1863.

Drapetetini: Lohse 1979: 200 [incorrect stem formation (ICZN 1999, Art. 29.3)].

###### Genus *Drapetes* Dejean, 1821

*Drapetes* Dejean, 1821: 34. Gender: masculine. Type species: *Elaterequestris* Fabricius, 1801: 244 (syn. of *Elatermordelloides* Host, 1790), by monotypy.

####### Subgenus Drapetes Dejean, 1821

*Drapetes* Dejean, 1821: 34. Gender: masculine. Type species: *Elaterequestris* Fabricius, 1801: 244 (syn. of *Elatermordelloides* Host, 1790: 298), by monotypy.

*Lissodes* Berthold, 1827: 335. Gender: masculine. Type species: *Elaterequestris* Fabricius, 1801: 244 (syn. of *Elatermordelloides* Host, 1790: 298), by subsequent designation (Smetana 2007: 46). Junior objective synonym of *Drapetes* Dejean, 1821.

*Paean* Gistel, 1848b: ix. Gender: masculine. Unnecessary replacement name for *Drapetes* Dejean, 1821.

*Draptetes*: Wolcott 1936: 214 [unavailable name, incorrect subsequent spelling not in prevailing usage].

####### Subgenus Neodrapetes Cobos, 1967

*Neodrapetes* Cobos, 1967: 311. Gender: masculine. Type species: *Drapetessellatus* Bonvouloir, 1859: 59, by original designation.

###### Genus *Hypochaetes* Bonvouloir, 1859

*Hypochaetes* Bonvouloir, 1859: 137. Gender: masculine. Type species: *Hypochaetessericeus* Bonvouloir, 1859: 139, by monotypy.

###### Genus *Lissomus* Dalman, 1824

*Lissomus* Dalman, 1824: 13. Gender: masculine. Type species: *Lissomuspunctulatus* Dalman, 1824: 14, by subsequent designation (Fleutiaux 1947b: 138).

*Cymbium*: Gerstaecker (1860: 136) [unavailable name, first proposed as a synonym and not made available subsequently (ICZN 1999, Art. 11.6)]

###### Genus *Osslimus* Calder, 1996

*Osslimus* Calder, 1996: 38. Gender: masculine. Type species: *Lissomusfreyi* Cobos, 1967: 323, by original designation.

###### Genus *Paradrapetes* Fleutiaux, 1895

*Paradrapetes* Fleutiaux, 1895: cccxci. Gender: masculine. Type species: *Paradrapetesvillosus* Fleutiaux, 1895: cccxci, **here designated**.

##### Tribe Protelaterini Schwarz, 1902c

Protelateridae Schwarz, 1902c: 365. Type genus: *Protelater* Sharp, 1877.

Sphaenelaterini Stibick, 1979: 179. Type genus: *Sphaenelater* Schwarz, 1902.

###### Genus *Anaspasis* Candèze, 1882

*Anaspasis* Candèze, 1882: 4. Gender: feminine. Type species: *Anaspasisfasciolata* Candèze, 1882: 5, by monotypy.

###### Genus *Austrelater* Calder & Lawrence, 1993

*Austrelater* Calder & Lawrence in Calder, Lawrence & Trueman, 1993: 1351. Gender: masculine. Type species: *Austrelatermacphersonensis* Calder in Calder, Lawrence & Trueman, 1993: 1354, by original designation.

###### Genus *Protelater* Sharp, 1877

*Protelater* Sharp, 1877: 482. Gender: masculine. Type species: *Protelaterelongatus* Sharp, 1877: 482, by subsequent designation (Hyslop 1921: 667).

###### Genus *Sphaenelater* Schwarz, 1902

*Geranus* Sharp, 1877: 480. Gender: masculine. Type species: *Geranuscrassus* Sharp, 1877: 480, by subsequent designation (Hyslop 1921: 646). Preoccupied by *Geranus* Bonaparte, 1854: 661 [Aves].

*Sphaenelater* Schwarz, 1902c: 365. Gender: masculine. Type species: *Sphaenelaternigricornis* Schwarz, 1902c: 365, by monotypy. Synonymised with *Geranus* Sharp, 1877 by Schwarz (1907: 286).

*Geraniella* Gourlay, 1950: 192. Gender: feminine. Unnecessary replacement name for *Geranus* Sharp, 1877.

###### Genus *Tunon* Arias-Bohart, 2013

*Tunon* Arias-Bohart, 2013: 161. Gender: masculine. Type species: *Tunonguinezi* Arias-Bohart, 2013: 164, by original designation.

###### Genus *Valdivelater* Lawrence & Arias, 2009

*Valdivelater* Lawrence & Arias, 2009: 320. Gender: masculine. Type species: *Valdivelaterkrahmeri* Lawrence & Arias, 2009: 322, by original designation.

#### Subfamily Oestodinae Hyslop, 1917

Oestodinae Hyslop, 1917: 251. Type genus: *Oestodes* LeConte, 1853.

Oestoidinae: Gurjeva 1974b: 68 [unavailable name, incorrect subsequent spelling not in prevailing usage].

##### Genus *Oestodes* LeConte, 1853

*Oestodes* LeConte, 1853: 424. Gender: masculine. Type species: *Elatertenuicollis* Randall, 1838: 14, by subsequent designation (Hyslop 1921: 659).

*Oestodus*: Smith 1900: 251 [unavailable name, incorrect subsequent spelling not in prevailing usage].

*Cestodes*: Brimley 1938: 167 [unavailable name, incorrect subsequent spelling not in prevailing usage].

##### Genus *Bladus* LeConte, 1861

*Bladus* LeConte, 1861: 171. Gender: masculine. Type species: *Eucnemisquadricollis* Say, 1839: 186, by subsequent monotypy in LeConte (1863: 48).

#### Subfamily Parablacinae Kundrata, Gunter, Douglas & Bocak, 2016

Parablacinae Kundrata, Gunter, Douglas & Bocak, 2016: 299. Type genus: *Parablax* Schwarz, 1906.

##### Genus *Metablax* Candèze, 1869

*Blax* Candèze, 1863: 200. Gender: masculine (ICZN 1999, Art. 30.1.4.2). Type species: *Elateracutipennis* White, 1846: 7, by subsequent designation (Hyslop 1921: 631). Preoccupied by *Blax* Koch, 1840: 359 [Collembola] and *Blax* J Thomson, 1860: 22 [Coleoptera: Cerambycidae].

*Metablax* Candèze, 1869: 122. Gender: masculine. Replacement name for *Blax* Candèze, 1863.

##### Genus *Ophidius* Candèze, 1863

*Ophidius* Candèze, 1863: 203. Gender: masculine. Type species: *Ophidiuselegans* Candèze, 1863: 204, by subsequent designation (Hyslop 1921: 659).

##### Genus *Parablax* Schwarz, 1906

*Parablax* Schwarz, 1906c: 368. Gender: masculine. Type species: *Metablaxtrisulcatus* Schwarz, 1903: 394 (syn. of *Parasaphesquinquesulcatus* Blackburn, 1900: 114), by subsequent designation (Hyslop 1921: 661).

##### Genus *Parasaphes* Candèze, 1882

*Parasaphes* Candèze, 1882: 101. Gender masculine (ICZN 1999, Art. 30.1.4.2). Type species: *Parasapheselegans* Candèze, 1882: 101, by monotypy.

##### Genus *Sharon* Arias-Bohart & Elgueta, 2015

*Sharon* Arias-Bohart & Elgueta, 2015: 2. Gender: feminine (erroneously treated as masculine in original description; the correct name of the type species is *Sharonamoena*). Type species: *Asaphesamoenus* Philippi, 1861: 743, by original designation.

##### Genus *Tasmanelater* Calder, 1996

*Tasmanelater* Calder, 1996: 250. Gender: masculine. Type species: *Tasmanelaterpelionensis* Calder, 1996: 255, by original designation.

##### Genus *Wynarka* Calder, 1986

*Wynarka* Calder, 1986: 111. Gender: neuter (ICZN 1999, Art. 30.2.3).Type species: *Wynarkasylvestre* Calder, 1986: 113, by original designation.

##### Genus *Xuthelater* Calder, 1996

*Xuthelater* Calder, 1996: 261. Gender: masculine. Type species: *Xuthelatermoppiensis* Calder, 1996: 265, by original designation.

#### Subfamily Physodactylinae Lacordaire, 1857

Physodactylides Lacordaire, 1857: 236. Type genus: *Physodactylus* Fischer von Waldheim, 1823.

Physodactilini: Schwarz 1897: 12 [unavailable name, incorrect subsequent spelling not in prevailing usage]

Physodactilinae: Fleutiaux 1919: 106 [unavailable name, incorrect subsequent spelling not in prevailing usage]

Physodactytinae: Fleutiaux 1935: 116 [unavailable name, incorrect subsequent spelling not in prevailing usage]

##### Genus *Dactylophysus* Fleutiaux, 1892

*Dactylophysus* Fleutiaux, 1892: 408. Gender: masculine. Type species: *Dactylophysuscapixabensis* Rosa, 2014: 249 (misidentification of *Dactylophysusmendax* Candèze, 1859 by Fleutiaux (1892)), fixed subsequently by Rosa (2014: 249) under ICZN (1999, Art. 70.3).

##### Genus *Physodactylus* Fischer von Waldheim, 1823

*Physodactylus* Fischer von Waldheim, 1823: 301. Gender: masculine. Type species: *Physodactylushenningii* Fischer von Walheim, 1823: 303, by monotypy.

*Drepanius* Perty, 1830: 24. Gender: masculine. Type species: *Drepaniusclavipes* Perty, 1830: 25, by monotypy. Synonymised with *Physodactylus* Fischer von Waldheim, 1823 by Laporte (1840: 254).

*Drepranius*: Schwarz 1907: 311 [unavailable name, incorrect subsequent spelling not in prevailing usage].

#### Subfamily Pityobiinae Hyslop, 1917

Pityobini Hyslop, 1917: 249. Type genus: *Pityobius* LeConte, 1853. Incorrect original stem formation, not in prevailing usage; see Bouchard et al. (2011).

Pytiobinae: Dolin (1968: 64) [unavailable name, incorrect subsequent spelling not in prevailing usage].

Pityobinae: Gurjeva (1974a: 103) [unavailable name, incorrect subsequent spelling not in prevailing usage].

Pityobiinae: Dolin (1975: 1629). Correct stem formation.

Pytiobiininae: Arias-Bohart and Elgueta 2012: 648 [unavailable name, incorrect subsequent spelling not in prevailing usage].

Pytiobiinini: Arias-Bohart and Elgueta 2012: 648 [unavailable name, incorrect subsequent spelling not in prevailing usage].

Pytiobiinae: Arias-Bohart and Elgueta 2015: 13 [unavailable name, incorrect subsequent spelling not in prevailing usage].

##### Genus *Pityobius* LeConte, 1853

*Pityobius* LeConte, 1853: 428. Gender: masculine. Type species: *Pityobiusanguinus* LeConte, 1853: 428, by monotypy.

*Calocerus* LeConte, 1853: 428. Gender: masculine. Type species: *Pityobiusanguinus* LeConte, 1853: 428, by monotypy. First proposed as a synonym of *Pityobius* LeConte, 1853. The name *Calocerus* LeConte, 1853 is available because it was treated as a senior homonym [of *Calocerus* Fauvel, 1891: 88; Coleoptera: Staphylinidae] before 1961 by Blackwelder (1952: 90) (ICZN 1999, Art. 11.6.1). First Reviser (ICZN 1999, Art. 24.2) found (*Pityobius* LeConte, 1853 vs. *Calocerus* LeConte, 1853) is Arnett (1962: 505). However, the specific name *niger* published by LeConte, 1853 is not available as it has never been used as valid or considered a homonym. The originally included species in *Calocerus* LeConte is that species listed by LeConte as valid, i.e., *Pityobiusanguinus* LeConte, 1853 (see ICZN 1999, Article 67.12).

*Pytiobius*: Schwarz 1906a: 192 [unavailable name, incorrect subsequent spelling not in prevailing usage].

##### Genus *Tibionema* Solier, 1851

*Tibionema* Solier, 1851: 30. Gender: neuter (erroneously used as feminine in original description). Type species: *Tibionemarufiventre* Solier, 1851: 31, by original designation.

#### Subfamily Subprotelaterinae Fleutiaux, 1920

Subprotelaterinae Fleutiaux, 1920: 99. Type genus: *Subprotelater* Fleutiaux, 1916.

##### Genus *Subprotelater* Fleutiaux, 1916

*Subprotelater* Fleutiaux, 1916b: 387. Gender: masculine. Type species: *Subprotelaterbakeri* Fleutiaux, 1916b: 387, by monotypy.

#### Subfamily Tetralobinae Laporte, 1840

Tetralobites Laporte, 1840: 230. Type genus: *Tetralobus* Lepeletier & Audinet-Serville, 1828.

##### Tribe Tetralobini Laporte, 1840

Tetralobites Laporte, 1840: 230. Type genus: *Tetralobus* Lepeletier & Audinet-Serville, 1828.

Phyllophoridae Hope, 1842: 73. Type genus: *Phyllophorus* Hope, 1842. Preoccupied by *Phyllophorus* Grube, 1840 [Echinodermata]; syn. of *Tetralobus* Lepeletier and Audinet-Serville, 1828. Permanently invalid (ICZN 1999, Art. 39), based on preoccupied type genus.

Piezophyllini Laurent, 1967: 85. Type genus: *Piezophyllus* Hope, 1842.

Tetralobilini: Costa, Casari-Chen & Vanin, 1992: 887 [incorrect stem formation (ICZN 1999, Art. 29.3)].

###### Genus *Neotetralobus* Girard, 1987

*Neotetralobus* Girard, 1987: 49. Gender: masculine. Type species: *Neotetralobusafricanus* Girard, 1987: 51, by monotypy.

###### Genus *Paratetralobus* Laurent, 1964

*Paratetralobus* Laurent, 1964: 220. Gender: masculine. Type species: *Tetralobushemirhipoides* Fleutiaux, 1919: 36, by original designation.

###### Genus *Pseudalaus* Laurent, 1967

*Pseudalaus* Laurent, 1967: 92. Gender: masculine. Type species: *Tetralobusdohrni* Candèze, 1882: 26, by original designation.

###### Genus *Pseudotetralobus* Schwarz, 1902

*Pseudotetralobus* Schwarz, 1902a: 210. Gender: masculine. Type species: *Pseudotetralobusdohrni* Schwarz, 1902a: 211, by monotypy.

###### Genus *Sinelater* Laurent, 1967

*Sinelater* Laurent, 1967: 94. Gender: masculine. Type species: *Tetralobusperroti* Fleutiaux, 1940: 107, by original designation.

###### Genus *Tetralobus* Lepeletier & Audinet-Serville, 1828

*Tetralobus* Lepeletier & Audinet-Serville, 1828: 594. Gender: masculine. Type species: *Elaterflabellicornis* Linnaeus, 1767: 651, by subsequent designation (Lacordaire 1857: 164).

####### Subgenus Dodecamerus Laurent, 1968

*Dodecamerus* Laurent, 1968: 328. Gender: masculine. Type species: *Tetralobusangolensis* Laurent, 1968: 328, by monotypy.

####### Subgenus Tetralobus Lepeletier & Audinet-Serville, 1828

*Tetralobus* Lepeletier & Audinet-Serville, 1828: 594. Gender: masculine. Type species: *Elaterflabellicornis* Linnaeus, 1767: 651, by subsequent designation (Lacordaire 1857: 164).

*Phyllophorus* Hope, 1842: 73. Gender: masculine. Type species: *Elatergigas* Fabricius, 1801: 221, by monotypy. Preoccupied by *Phyllophorus* Grube, 1840: 38 [Echinodermata].

*Charitophyllus* Lacordaire, 1857: 165. Gender: masculine. Replacement name for *Phyllophorus* Hope, 1842. Synonymised with *Tetralobus* Lepeletier & Audinet-Serville, 1828 by Candèze (1857: 366).

*Tetralobius*: Froggatt 1917: 894 [unavailable name, incorrect subsequent spelling not in prevailing usage].

*Tetralobes*: Kubaczkova and Kundrata 2017: 161 [unavailable name, incorrect subsequent spelling not in prevailing usage erroneously attributed to Dolin (ICZN 1999, Art. 33.3)].

##### Tribe Piezophyllini Laurent, 1967

Piezophyllini Laurent, 1967: 96. Type genus: *Piezophyllus* Hope, 1842.

###### Genus *Piezophyllus* Hope, 1842

*Piezophyllus* Hope, 1842: 76. Gender: masculine. Type species: *Tetralobusrobustus* Hope, 1842: 75 (syn. of *Tetralobusmacrocerus* Laporte, 1838: 12), by original designation.

*Cladocerus*: Harold 1869: 1509 [unavailable name, first proposed as a synonym and not made available subsequently (ICZN 1999, Art. 11.6)]. If found to be available then preoccupied by *Cladocerus* Rafinesque, 1819: 429 [Coelenterata].

*Coresus*Harold 1869: 1509. Gender: masculine. Type species: *Tetralobusmacrocerus* Laporte, 1838: 12, by monotypy. Originally proposed as a synonym of *Piezophyllus* Hope, 1842 and subsequently treated as valid.

*Dido* Arnett, 1955: 600. Gender: feminine. Type species: *Tetralobusmacrocerus* Laporte, 1838: 12, by original designation. Junior objective synonym of *Coresus*Harold 1869.

*Hopelater* Laurent, 1967: 99. Gender: masculine. Type species: *Piezophyllusspencei* Hope, 1842: 76, by original designation. Synonymised with *Piezophyllus* Hope, 1842 by Costa et al. (1994: 120).

*Piezophilus*: Costa, Casari-Chen & Vanin, 1992: 879 [unavailable name, incorrect subsequent spelling not in prevailing usage].

*Piezophylus*: Costa, Casari-Chen and Vanin 1992: 879 [unavailable name, incorrect subsequent spelling not in prevailing usage].

## Footnotes

The gender of this genus has been contentious in the past. Under the provisions of Art. 30 of the Code (ICZN 1999), and because of the lack of any etymology from Laporte (1838), it may correspond to the Greek word Λάκων (a Laconian or Spartan man), transliterated without changes as Lacon following the Art. 30.1.2. In the Greek dictionaries, this word is masculine, so the genus is masculine too, as well as all the genera having – lacon as their last component.

Latinised form of the Greek noun τριήρης (a trireme, in allusion to the dilated tarsi of its type species). According to the provisions of the ICZN (1999, Art. 30.1.2), if the noun is feminine, the genus is also feminine. The specific name ramitarsus can be considered a noun in apposition.
